# IFITM1 enhances nonenveloped viral RNA replication by facilitating cholesterol transport to the Golgi

**DOI:** 10.1371/journal.ppat.1011383

**Published:** 2023-05-30

**Authors:** Kumiko ISHIKAWA-Sasaki, Takayuki Murata, Jun Sasaki

**Affiliations:** Department of Virology, Fujita Health University School of Medicine, Kutsukakecho, Toyoake, Aichi, Japan; University of Maryland, UNITED STATES

## Abstract

Aichi virus (AiV), a small non-enveloped RNA virus, hijacks the endoplasmic reticulum (ER)–Golgi cholesterol transport machinery to form cholesterol-rich replication sites originating from Golgi membranes. Interferon-induced transmembrane proteins (IFITMs) are antiviral restriction factors, whose involvement in intracellular cholesterol transport is suggested. Here, we describe the roles of IFITM1 in cholesterol transport that affect AiV RNA replication. IFITM1 stimulated AiV RNA replication and its knockdown significantly reduced the replication. In replicon RNA-transfected or infected cells, endogenous IFITM1 localized to the viral RNA replication sites. Further, IFITM1 interacted with viral proteins and host Golgi proteins, ACBD3, PI4KB, OSBP, which constitute the replication sites. When overexpressed, IFITM1 localized to the Golgi as well as endosomes, and this phenotype was also observed for endogenous IFITM1 early in AiV RNA replication, leading to the distribution of cholesterol at the Golgi-derived replication sites. The pharmacological inhibition of ER–Golgi cholesterol transport or endosomal cholesterol export impaired AiV RNA replication and cholesterol accumulation at the replication sites. Such defects were corrected by expression of IFITM1. Overexpressed IFITM1 facilitated late endosome–Golgi cholesterol transport without any viral proteins. In summary, we propose a model in which IFITM1 enhances cholesterol transport to the Golgi to accumulate cholesterol at Golgi-derived replication sites, providing a novel mechanism by which IFITM1 enables efficient genome replication of non-enveloped RNA virus.

## Author summary

Aichi virus (AiV), a non-enveloped RNA virus, is a pathogen associated with acute gastroenteritis in humans. Previously, we indicated that AiV RNA replication requires cholesterol accumulation at the replication sites by cholesterol transport pathway involving oxysterol-binding protein (OSBP). The interferon-inducible transmembrane proteins (IFITMs) are antiviral factors that inhibits infectious entry of many enveloped RNA viruses, and IFITM3 is suggested to be negatively involved in OSBP-mediated cholesterol transport. When we investigated the effects of IFITMs on AiV genome replication, we unexpectedly found that IFITM1 enhanced the replication. When overexpressed, IFITM1 localized to the Golgi in addition to endosomes. Similarly, IFITM1 localized to both organelles during AiV genome replication. We also confirmed that IFITM1 is involved in cholesterol trafficking to the Golgi. By utilizing this IFITM1’s ability, AiV accumulated cholesterol at the Golgi-originating specialized compartment where AiV replicates through interaction among AiV proteins, Golgi proteins, and IFITM1, resulting in AiV replication enhancement. Our findings may contribute to the understanding unknown functions of IFITM1 and its effects on virus infection.

## Introduction

Aichivirus (AiV) is a small nonenveloped RNA virus that is a member of the genus *Kobuvirus* of the family *Picornaviridae* [1], to which poliovirus, coxsackieviruses, and numbered enteroviruses belong. AiV, first isolated in 1989 in Aichi, Japan, is distributed worldwide, and, although not often, has been suggested to be the cause of gastroenteritis [2,3]. The positive-sense, single-stranded RNA genome of AiV is approximately 8.3 kb long and has a single, large open reading frame encoding a polyprotein that is posttranslationally processed by the virus-encoded protease 3C into individual proteins. The AiV nonstructural membrane-associated proteins, 2B, 2BC, 2C, 3A, and 3AB constitute viral RNA replication sites and are essential for AiV RNA replication [4–6]. These viral proteins all bind to the Golgi protein acyl-coenzyme A binding domain containing 3 (ACBD3), which interacts with phosphatidylinositol 4-kinase IIIβ (PI4KB) to form a viral protein/ACBD3/PI4KB complex, enhancing PI4KB-dependent phosphatidylinositol 4-phosphate (PI4P) production at the replication sites [4,5].

AiV also utilizes OSBP, a PI4P-binding cholesterol transfer protein, for viral RNA replication [6]. OSBP binds to vesicle-associated membrane protein-associated protein A and B (VAPA and VAPB) on the endoplasmic reticulum (ER) and to PI4P on the replication sites originating from the Golgi, and shuttles PI4P and cholesterol between the ER and Golgi-derived replication sites, resulting in cholesterol accumulation at the replication sites [6]. The lipid phosphatase Sac1, which dephosphorylates PI4P transported from the replication sites to the ER, is also a component of the OSBP-involving cholesterol transport pathway. The cholesterol recruitment to the replication sites is essential to viral RNA replication [6].

A viral infection activates the host innate immune response for viral clearance. Interferons (IFNs), which play a major role in innate immunity, induce the expression of IFN-stimulated genes (ISGs) to mediate antiviral activity [7]. Among the ISGs, genes encoding IFN-induced transmembrane proteins (IFITMs) are induced by both type I and II IFNs and conserved across vertebrates [8,9]. IFITM1, IFITM2, and IFITM3 in humans are expressed ubiquitously in most cell types, and partially colocalize with endosome markers [10–13]. IFITM1 is also localized to the plasma membrane [11,14]. IFITMs restrict the infection of many highly pathogenic enveloped RNA viruses, including influenza A virus, flaviviruses, filoviruses, severe acute respiratory syndrome coronavirus (SARS-CoV), SARS-CoV-2 [15–18]. Conversely, IFITMs promote the infection of human coronavirus OC43 and some human herpesviruses [19–22]. In this regard, the recent report showed that endogenous IFITMs are cofactors for SARS-CoV-2 infection [23]. While the exact role of IFITMs remain to be established, experimental evidence suggests involvement of IFITMs in cholesterol transport, which IFITM3 perturbs cholesterol metabolism in endosomes by binding to VAPA in competition with OSBP [10]. Both VAPA and OSBP are host factors required for AiV RNA replication, and cholesterol transport mediated by their interaction is essential for the replication. Thus, this study investigated the impact of IFITMs on viral RNA replication.

Contrary to expectations, we found that IFITM1, but not IFITM2 and 3, had an enhancing effect on AiV RNA replication. IFITM1 localized to the viral RNA replication sites, where viral and host Golgi proteins required for replication were associated. We showed that IFITM1, when overexpressed, localizes not only to the endosomes but also to the Golgi in non-infected cells. In addition, overexpressed IFITM1 led to cholesterol distribution in the Golgi and the Golgi-derived replication sites in the absence and in the presence of viral proteins, respectively. In AiV RNA replicating cells, when viral protein expression was still low, endogenous IFITM1 colocalized with endosomal and Golgi markers and cholesterol. Our results suggest that AiV hijacks an IFITM1-mediated cholesterol transport pathways to the Golgi, resulting in cholesterol accumulation at the Golgi-derived replication sites.

## Results

### IFN-α inhibits AiV RNA replication

**Fig 1:**
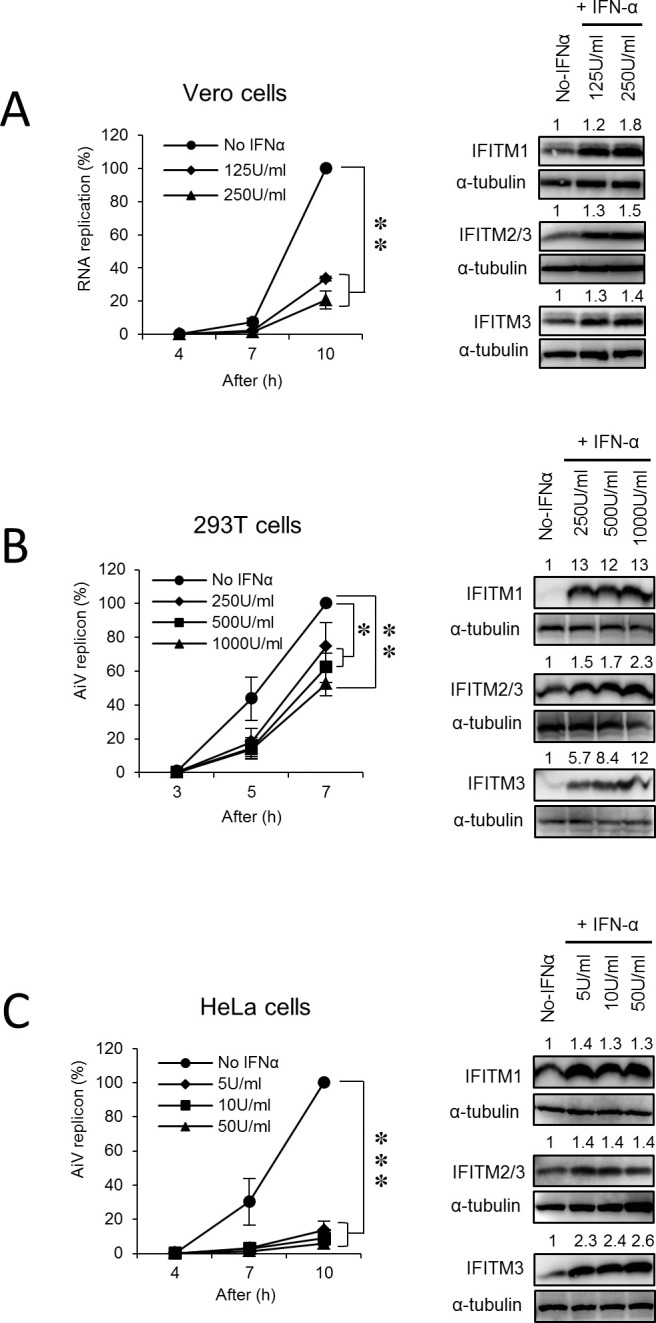
Effects of IFN-α on AiV RNA replication. Vero (A), 293 (B), and HeLa (C) cells were incubated with the indicated concentrations of IFN-α for 24 h and then transfected with AiV replicon RNA. At the indicated time points after transfection, cell lysates were harvested, and luciferase activity was measured. Each right panel represents the protein levels of IFITM1, IFITM2, or IFITM3 by immunoblotting for cell lysates harvested from experiments shown in the left panels. Data are the means ±SD of at least three independent experiments. *, *P* < 0.05; **, *P* < 0.001; ***, *P* < 0.0001.

To examine the effect of type I IFN (IFN-α) on AiV RNA replication, Vero cells, a standard cell line to grow AiV, were treated with the indicated concentrations of IFN-α for 24 h and then transfected with AiV replicon RNA containing a firefly luciferase gene, which allowed us to assess replication levels by a luciferase assay. At 10 h after transfection, viral RNA replication in IFN-α-treated cells was clearly reduced compared to nontreated cells (Fig 1A, left). IFN-α treatment at both concentrations resulted in an increased expression of IFITMs (Fig 1A, right). To investigate whether IFN sensitivity to AiV RNA replication is cell type specific, similar tests were conducted on 293T and HeLa cells. In 293T cells, replication was inhibited by ~50% in IFN-α-treated cells at high levels (1000 U/ml IFN-α) (Fig 1B) but was blocked significantly in HeLa cells treated with only 5 U/ml IFN-α (Fig 1C). In both cells, increased expression levels of IFITMs were confirmed at all IFN-α concentrations tested (Fig 1B and 1C). These observations indicate that AiV RNA replication is sensitive to IFN-α-induced antiviral response in a variety of cell lines.

### IFITM1 enhances AiV RNA replication

**Fig 2:**
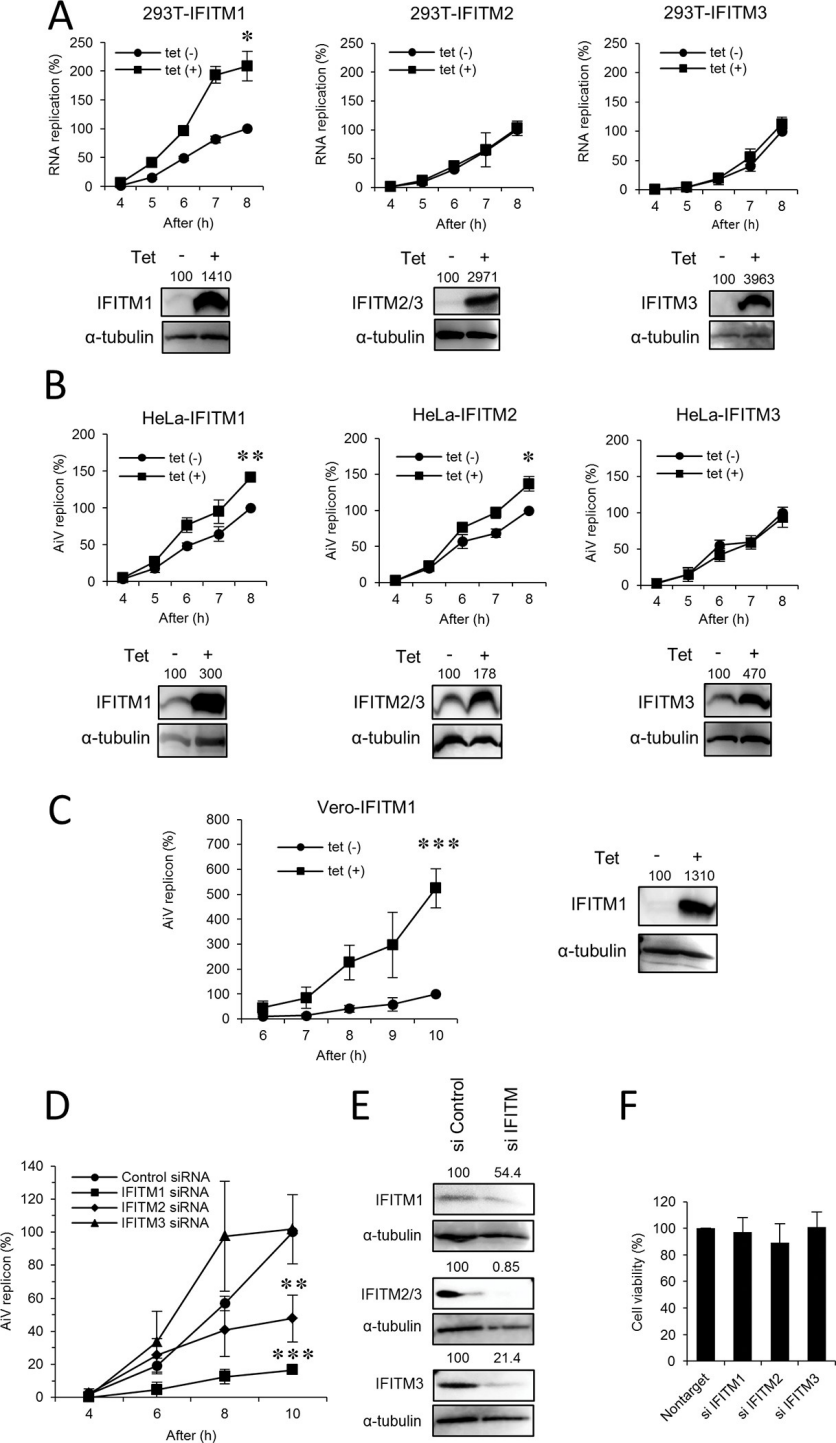
IFITM1 enhances AiV RNA replication in multiple cell lines. (A–C) 293 (A), HeLa (B), or Vero (C) cell lines stably expressing tetracycline (Tet)-inducible IFITM1, IFITM2 or IFITM3 were cultured with or without Tet for 72 h and then transfected with AiV replicon RNA. Cell lysates were harvested at the indicated time points after transfection and then assayed for luciferase activity. The peak activity obtained for cells in the absence of Tet was taken as 100%. Tet-induced IFITM1, IFITM2, or IFITM3 protein expression in each cell line was detected by Western blotting (bottom panels of (A) and right panel of (B) or right panel of (C)). (D) HeLa cells were transfected with the control, IFITM1, IFITM2, or IFITM3 siRNA for 72 h and then transfected with replicon RNA. At the indicated time points after replicon RNA transfection, cell lysates were harvested and subjected to the luciferase assay. The maximum value obtained for cells treated with control siRNA was taken as 100%. (E) IFITM1, IFITM2, or IFITM3 knockdown was confirmed by Western blotting. (F) Cell viability was determined by the CellTiter-Glo assay. Data are the means ± SD of at least three independent experiments. *, *P* < 0.05; **, *P* < 0.001; ***, *P* < 0.0001.

To confirm whether the suppression of AiV RNA replication by IFN-α is due to IFITMs among ISGs produced in response to IFN-α, we generated stable tetracycline (Tet)-inducible cell lines expressing IFITM1, IFITM2, or IFITM3 using 293T and HeLa cells, which showed a contrasting effect, as depicted in Fig 1B and 1C, suggesting response to IFN-α in cell-dependent manner. Cells were cultured with or without Tet for 72 h, and then transfected with the replicon RNA. At 8 h after transfection, contrary to expectations, Tet treatment of 293T-IFITM1, but not 293T-IFITM2 or 293-IFITM3 cells, showed a twofold increase in AiV RNA replication (Fig 2A). Similarly, a modest replication enhancement was also observed in HeLa-IFITM1 and HeLa-IFITM2 cells (Fig 2B). Furthermore, there was a fivefold enhancement of replication in Vero-IFITM1 cells (Fig 2C). Western blot analysis showed the Tet-induced expression of each IFITM in different cell types (Fig 2A–2C). These results imply that IFITM1 functions to promote AiV RNA replication in a cell type-independent manner.

To verify the role of IFITM1 in AiV RNA replication, siRNA knockdown assays in HeLa cells were carried out. As shown in Fig 2D, the depletion of IFITM1 or IFITM2 inhibited replication by 84% or 52%, respectively, compared to the control, although the knockdown efficiency of IFITM1 was lower than that of IFITM2 or IFITM3 (Fig 2E). In contrast, IFITM3 silencing did not affect replication at 10 h after transfection. The decrease in each IFITM expression by siRNA did not change the cell viability (Fig 2F). These data suggest that IFITM1 plays a role in upregulation of AiV RNA replication.

### IFITM1 interacts with AiV proteins 2B, 2BC, 2C, 3A, and 3AB

**Fig 3:**
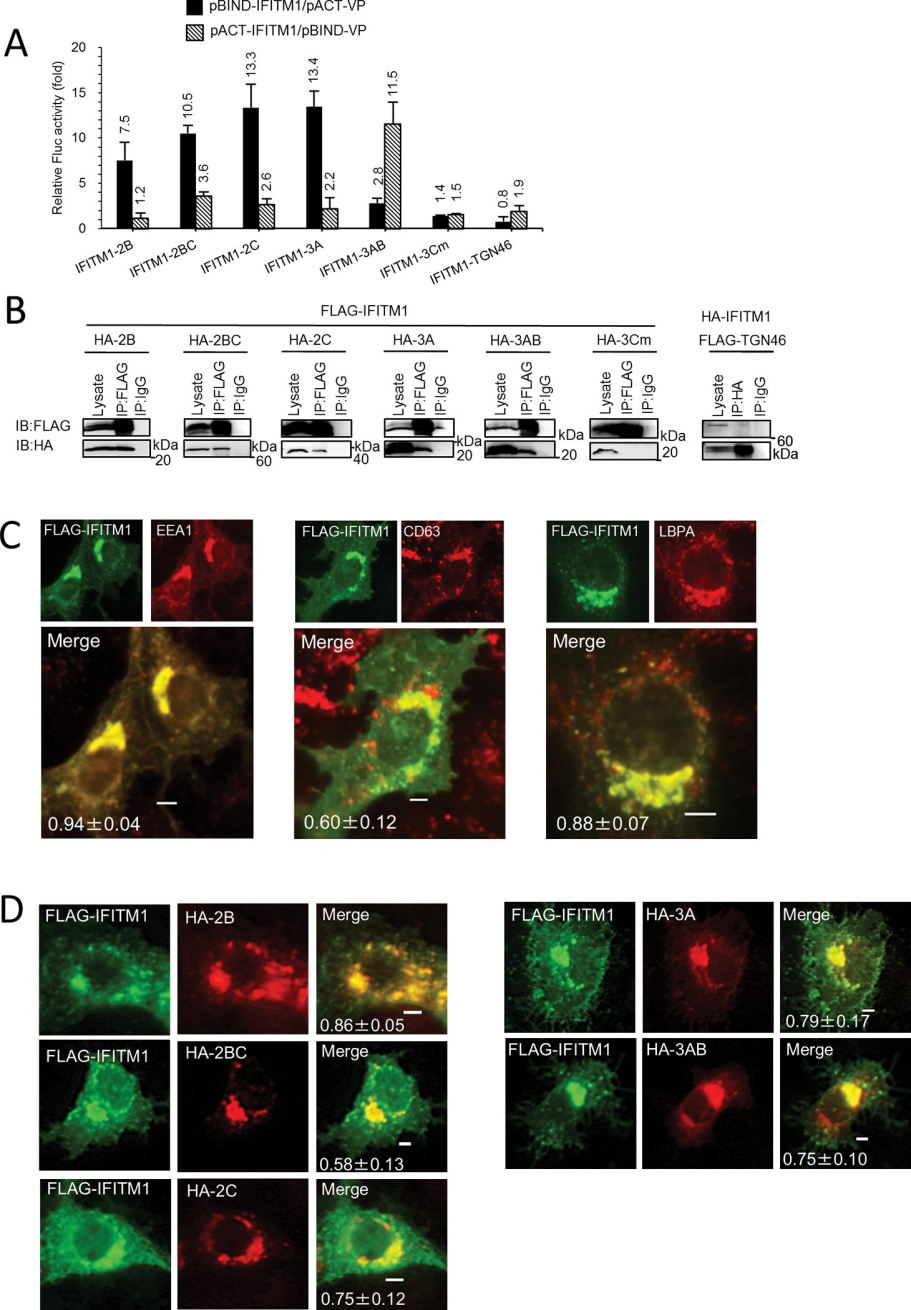
Interaction and colocalization of IFITM1 and AiV proteins, 2B, 2BC, 2C, 3A, and 3AB. (A) A mammalian two-hybrid assay was performed by transfecting Vero cells with the indicated combinations of pACT and pBIND plasmids along with the pG5luc firefly luciferase reporter plasmid. At 48 h after transfection, the normalized firefly luciferase activity (firefly luciferase activity/Renilla luciferase activity) was measured and represented as fold of the control activity, which was obtained by combination with the empty pACT or empty pBIND plasmid. Data are the mean ± SD of at least three independent experiments. (B) 293T cells were transfected for 24 h with FLAG-tagged IFITM1 and HA-tagged 3Cm, 2B, 2BC, 2C, 3A, or 3AB, or HA-tagged IFITM1 and FLAG-tagged TGN46 expression plasmids, as indicated, followed by coimmunoprecipitation (IP) with an anti-FLAG, -HA or control IgG antibody. The resulting immunoprecipitates and whole-cell lysates were subjected to immunoblotting (IB) with anti-FLAG and anti-HA antibodies. (C) Vero cells were transfected with FLAG-IFITM1. At 24 h after post-transfection, the cells were fixed and double stained with anti-IFITM and anti-EEA1, anti-CD63, or anti-LBPA antibodies, as indicated. Pearson correlation coefficient analyses for data were obtained from ≥10 cells. Correlation coefficients are presented as the mean and standard deviation. (D) Vero cells was transfected with FLAG-IFITM1 and HA-2B, HA-2BC, HA-2C, HA-3A, or HA-3AB. At 24 h after transfection, cells were fixed and stained with anti-FLAG and anti-HA antibodies. Bars, 4 μm. Pearson correlation coefficient analyses for data were obtained from 4–8 cells.

The above results led us to examine the interactions between IFITM1 and AiV proteins 2B, 2BC, 2C, 3A, and 3AB, which constitute the AiV replication sites [4–6]. A mammalian two-hybrid (M2H) assay in Vero cells was first carried out by coexpression of a DNA-binding domain (from pBIND)-fused IFITM1 or viral protein and a transcription activation domain (from pACT)-fused viral protein or IFITM1, and the binding between them was detected as firefly luciferase activity normalized to *Renilla* luciferase activity. IFITM1 interacted with 2B, 2BC, 2C, 3A, and 3AB (7.5- to 13.4-fold increases in luciferase activity compared to the control), but not with 3Cm, an inactivated mutant of the AiV 3C protease [24], or TGN46, an integral membrane protein (Fig 3A).

To confirm the interactions identified by the M2H analyses, coimmunoprecipitation assay was performed. 293T cells were transfected with plasmids expressing FLAG-tagged IFITM1 and HA-tagged 2B, 2BC, 2C, 3A, 3AB, or 3Cm. The combination of FLAG-tagged TGN46 and HA-tagged IFITM1 was transfected as the control, since we failed to express HA-tagged TGN46 for unknown reasons. Then proteins were immunoprecipitated with an anti-FLAG, -HA or control IgG antibody. As shown in Figs 3B and S2A, IFITM1 was coimmunoprecipitated with 2B, 2BC, 2C, 3A, or 3AB, but not with 3Cm or TGN46. These results indicate that IFITM1 binds to AiV replication proteins 2B, 2BC, 2C, 3A, and 3AB.

In addition, the intracellular localization of IFITM1 and the viral proteins were determined by an immunofluorescence assay with cell permeabilization treatment. When expressed alone in Vero cells, FLAG-IFITM1 partially colocalized with early (EEA1) and late endosomal (CD63 and LBPA) markers (Fig 3C), as reported previously [10,11,25,26]. Similar early and late endosomal localization of IFITM1 was observed when IFITM1 was expressed by Tet induction in Vero-IFITM1 and HeLa-IFITM1 cells (S1 Fig). FLAG-IFITM1 co-overexpression with HA-2B, HA-2BC, HA-2C, HA-3A, or HA-3AB resulted in their partial colocalization in the areas with higher fluorescence intensity (Fig 3D). This data may support interactions of IFITM1 with these viral proteins.

### Overexpressed IFITM1 interact with the Golgi proteins ACBD3, PI4KB, and OSBP

**Fig 4:**
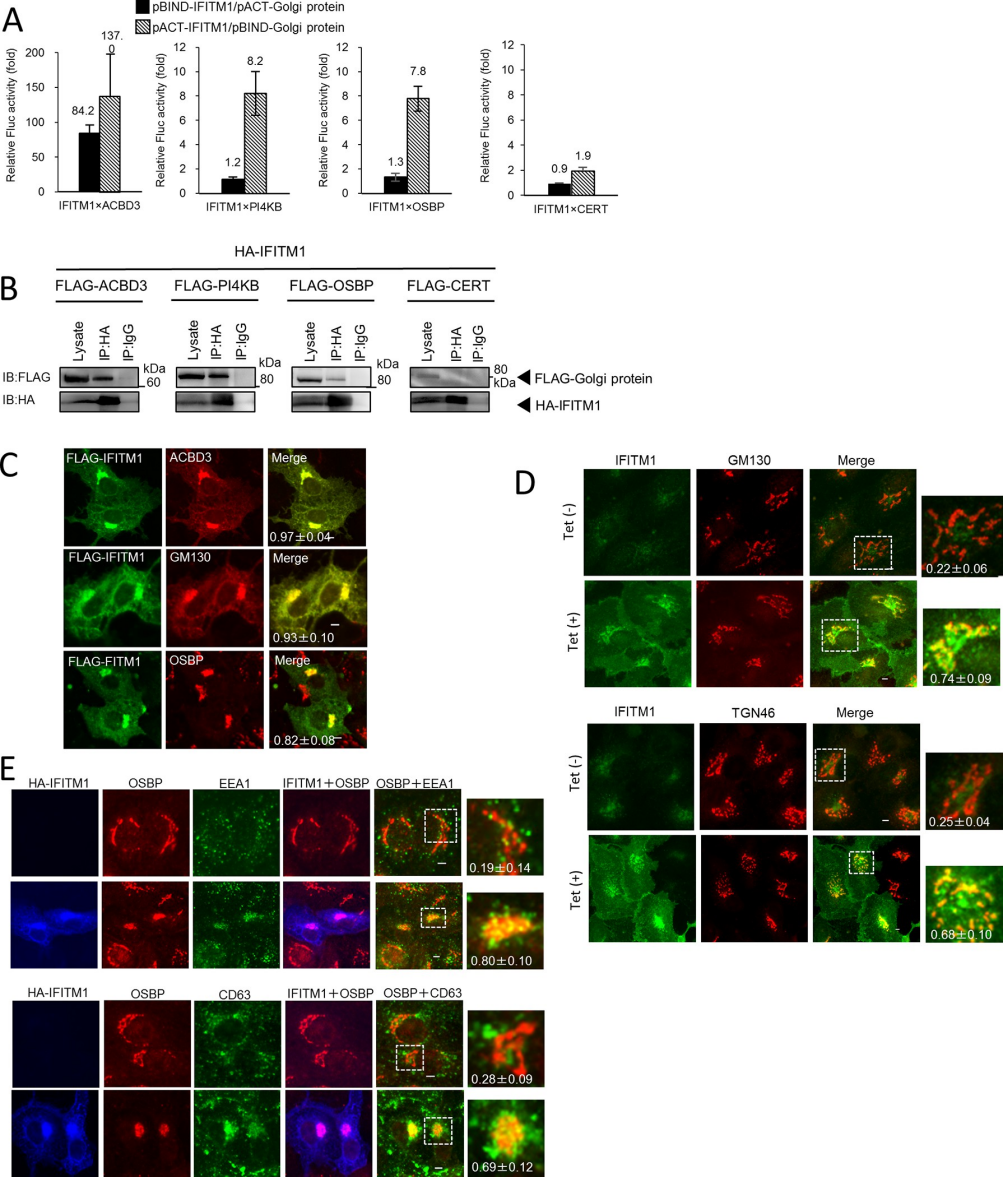
Subcellular localization analysis of overexpressed IFITM1. (A) A mammalian two-hybrid analysis was carried out to examine interactions between IFITM1 and ACBD3, PI4KB, OSBP, or CERT, and the results are shown as described in Fig 3A. Data are the mean ± SD of at least three independent experiments. (B) HA-tagged IFITM1 and FLAG-tagged ACBD3, PI4KB, OSBP, or CERT were cotransfected into 293T cells, and the cell lysates were subjected to immunoprecipitation with anti-HA antibody or control IgG. The resulting immunocomplexes and whole-cell lysates were detected by anti-FLAG and anti-HA antibodies. (C) Vero cells were transfected with FLAG-IFITM1. At 24 h, the cells were labeled with anti-FLAG and anti-ACBD3 (top), anti-PI4KB (middle), or anti-OSBP (bottom) antibodies. (D) Vero IFITM1 cells were incubated with Tet (−) or Tet (+) for 72 h, and the cells were fixed and double stained with the indicated antibodies. (E) Vero cells were transfected with HA-IFITM1. At 24 h, the cells were labeled using anti-HA, anti-OSBP and anti-EEA1, or anti-CD63 antibodies. Bars, 4 μm. Pearson correlation coefficient analyses for data were obtained from ≥10 cells. Correlation coefficients are presented as the mean and standard deviation (C-E).

Next, we examined whether IFITM1 interacts with Golgi proteins ACBD3, PI4KB, and OSBP, which are host factors essential to AiV RNA replication, and localize to the replication sites, together with AiV proteins 2B, 2BC, 2C, 3A, and 3AB [4–6]. As shown in Fig 4A, M2H analyses showed that IFITM1 interactions with ACBD3, PI4KB, and OSBP caused 84.2-, 8.2-, and 7.8-fold increases, respectively, in luciferase activity. On the other hand, the increased luciferase activity was only a 1.9-fold increase for binding to CERT, which localizes to the Golgi but is not recruited to the viral RNA replication sites [6], suggesting that the interactions of IFITM1 with ACBD3, PI4KB, and OSBP are specific. This was further confirmed by coimmunoprecipitation of IFITM1 with ACBD3, PI4KB, or OSBP, but not with CERT or TGN46 (Figs 3B, 4B, and S2B).

### Intracellular localization of overexpressed IFITM1

Previous reports and our observations indicate endosomal localization of IFITM1 [10–12] (Figs 3C and S1). However, IFITM1 was shown to bind to the Golgi proteins (Fig 4A and 4B). Therefore, we examined the intracellular localization of IFITM1 together with Golgi markers.

Fig 4C shows that transiently overexpressed FLAG-IFITM1 colocalized with endogenous ACBD3, GM130, or OSBP. Likewise, Tet-induced overexpression of IFITM1 also localized to the Golgi compartment, as evidenced by colocalization with endogenous GM130 or TGN46 (a trans-Golgi marker) (Fig 4D), as opposed to endogenous IFITM1 did not colocalized with these Golgi proteins as observed in Tet-uninduced cells. Next, we visualized the relationship between Golgi and endosomal localization in IFITM1 overexpression. As shown in Fig 4E, transiently overexpressed HA-IFITM1 colocalized with both OSBP and EEA1or CD63 in the juxtanuclear compartment (Fig 4E). Taken in combination, these results suggest that overexpressed IFITM1 targets not only to endosomes but also to the Golgi or that overexpressed IFITM1 induces proximity between the two organelles.

### Endogenous IFITM1 is localized to AiV replication sites

**Fig 5:**
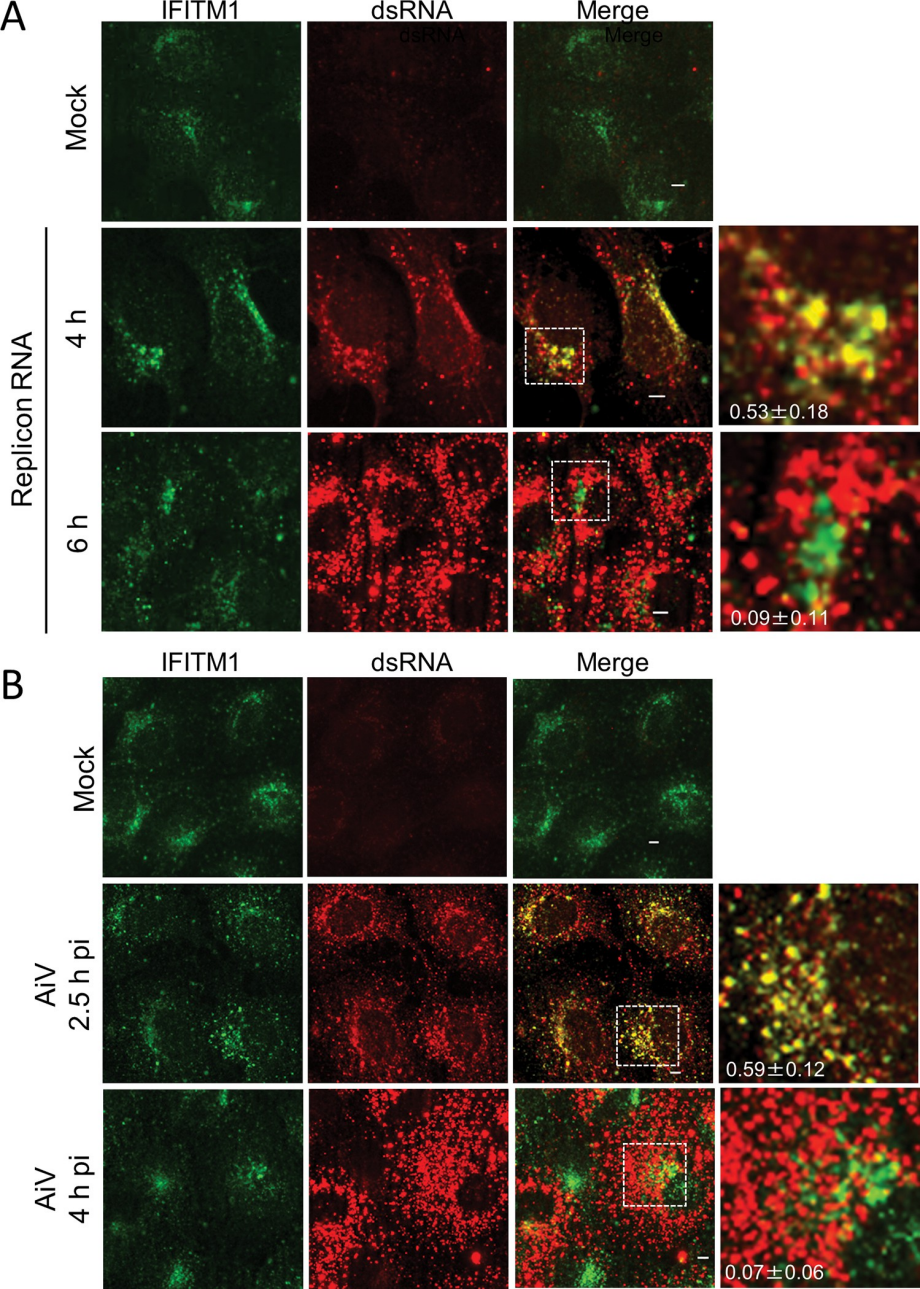
Endogenous IFITM1 localizes to the replication sites during the early stages of infection. (A) Vero cells were mock electroporated or electroporated with replicon RNA. After 4 or 6 h, the cells were fixed and stained with the indicated antibodies. (B) Vero cells were mock-infected or infected with AiV. After 2.5 or 4 h, the cells were fixed and stained with the indicated antibodies. Bars, 4 μm. Pearson correlation coefficient analyses for data were obtained from ≥10 cells. Correlation coefficients are presented as the mean and standard deviation (A and B).

To confirm whether IFITM1 is present at the AiV RNA replication sites, we examined the localization of endogenous IFITM1 and AiV RNA. Vero cells were mock-electroporated or electroporated with AiV replicon RNA, and at indicated time points, the cells were stained with antibodies against IFITM1 and double-stranded RNA, a marker for replicating viral RNA (Fig 5A). At 4 h after transfection, endogenous IFITM1 partially colocalized with dsRNA (Fig 5A Replicon RNA; 4 h). This colocalization was not seen as RNA replication progressed, as evidenced by the high fluorescence intensity of dsRNA at 6 h after transfection (Fig 5A, Replicon RNA; 6 h).

We also observed colocalization of endogenous IFITM1 and dsRNA in AiV-infected cells at 2.5 h post-infection but not at 4 h post-infection (Fig 5B), suggesting that endogenous IFITM1 localizes to viral replication sites early in infection.

### Endogenous IFITM1 localizes to Golgi-derived replication sites at an early state of AiV RNA replication

**Fig 6:**
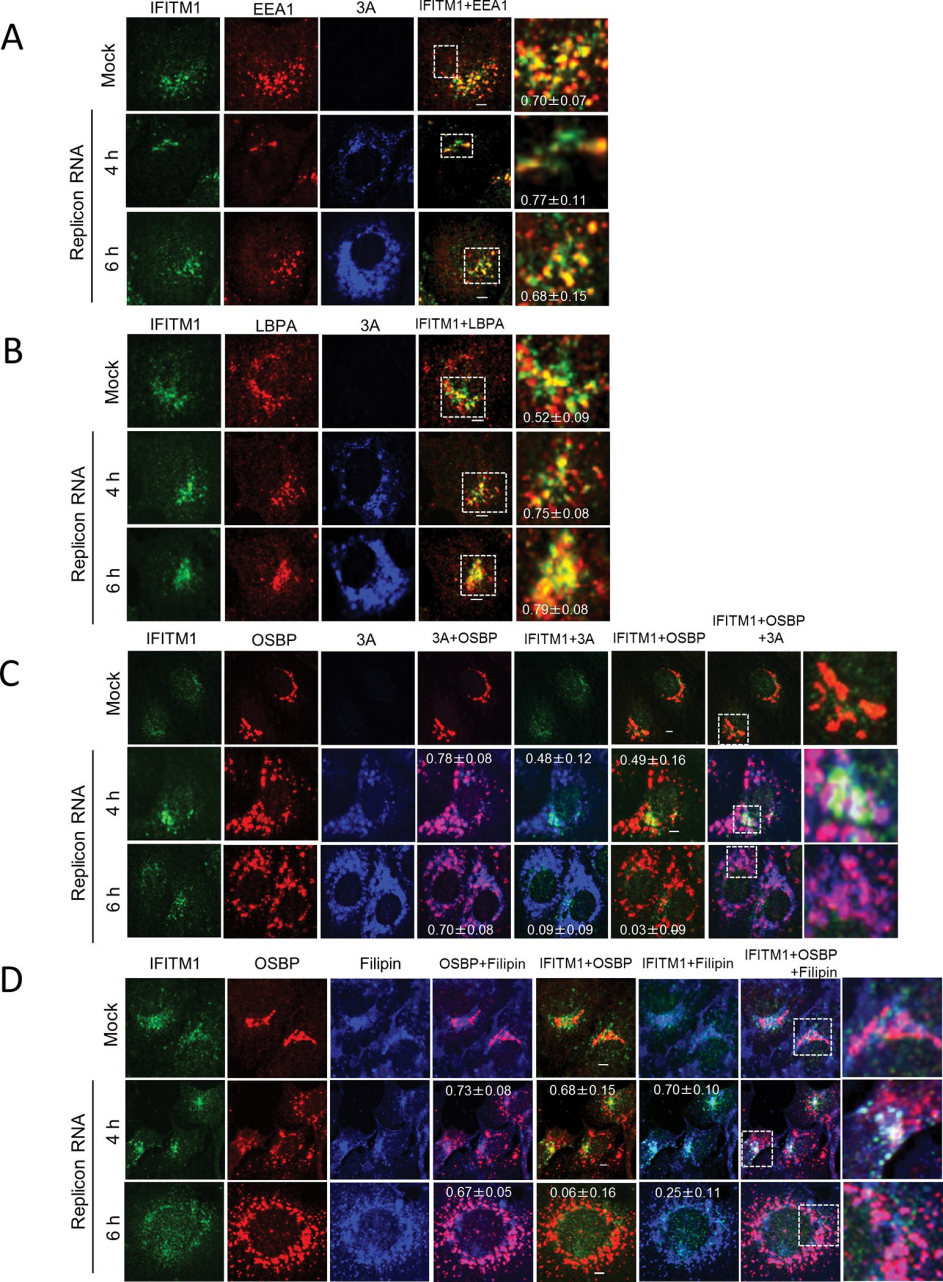
Endogenous IFITM1 localizes to both endosomes and the Golgi in cells with low 3A expression. (A–D) Vero cells were mock electroporated or electroporated with replicon RNA. After 4 or 6 h, the cells were fixed and stained using the indicated antibodies or filipin III (for cholesterol staining). Bars, 4μm. Pearson correlation coefficient analyses for data were obtained from ≥10 cells. Correlation coefficients are presented as the mean and standard deviation.

AiV replication sites are originated from the Golgi [4–6]. Given that endogenous IFITM1 localizes to the AiV RNA replication sites, we analyzed the localization of endogenous IFITM1 in AiV replicon replicating cells along with Golgi or endosomal markers. Vero cells electroporated with mock or replicon RNA were tested for colocalization of endogenous IFITM1 with EEA1, LBPA, or OSBP (Fig 6). In mock-transfected cells, endogenous IFITM1 partially colocalized with EEA1 (Fig 6A, Mock) and LBPA (Fig 6B, Mock), but not with OSBP (Fig 6C, Mock). On the contrary, at 4 h after transfection of replicon RNA, endogenous IFITM1 partially colocalized with OSBP in addition to EEA1 and LBPA in cells with low expression of 3A (Fig 6A–6C, Replicon RNA; 4 h). The colocalization of IFITM1, OSBP, and 3A indicates that IFITM1 localizes to the Golgi-derived replication site. We also confirmed the colocalization of OSBP with EEA1 or CD63 at low expression of 3A (S3 Fig, Replicon RNA). As expression of 3A increased at 6 h after transfection, the Golgi targeting of IFITM1 was lost (Fig 6C, Replicon RNA; 6 h), while IFITM1 remained in endosomes, as observed by the partially colocalization with EEA1 or LBPA. (Fig 6A and 6B, Replicon RNA; 6 h). These results suggest that endogenous IFITM1 can localize to Golgi-derived replication sites as well as to endosomes early in AiV RNA replication, but cannot contact with the Golgi as replication proceeds.

Previous studies have suggested the involvement of IFITM in cholesterol metabolism [10]. Thus, we additionally sought to confirm that cholesterol accumulates when IFITM1 localizes to Golgi-derived replication sites. The cholesterol distribution was determined using filipin, a fluorescent cholesterol-binding probe. As depicted in Fig 6D, IFITM1 colocalized with the Golgi protein OSBP, a marker of the replication sites, at 4 h after transfection as in Fig 6C, and also colocalized with filipin (Fig 6D, Replicon RNA; 4 h). Conversely, IFITM1 could not maintain colocalization with OSBP and filipin at 6 h after transfection, when the replication was enhanced and viral protein expression was increased (Fig 5D, Replicon RNA; 6 h), suggesting that IFITM1 is involved in the distribution of cholesterol at the replication sites during early genome replication.

### IFITM1 rescues AiV replication inhibition by cholesterol transport inhibitors

Based on the above results (Figs 4A, 4B, S4A, and S4B) and the finding that IFITM1 associates with OSBP and the ER proteins VAPA, VAPB, and SAC1 [10], we hypothesized that IFITM1 is involved in OSBP-mediated cholesterol transport. To verify this, we tested whether IFITM1 could counteract the antiviral effect of 25-HC that inhibit AiV RNA replication by blocking OSBP-mediated cholesterol transport [6].

**Fig 7:**
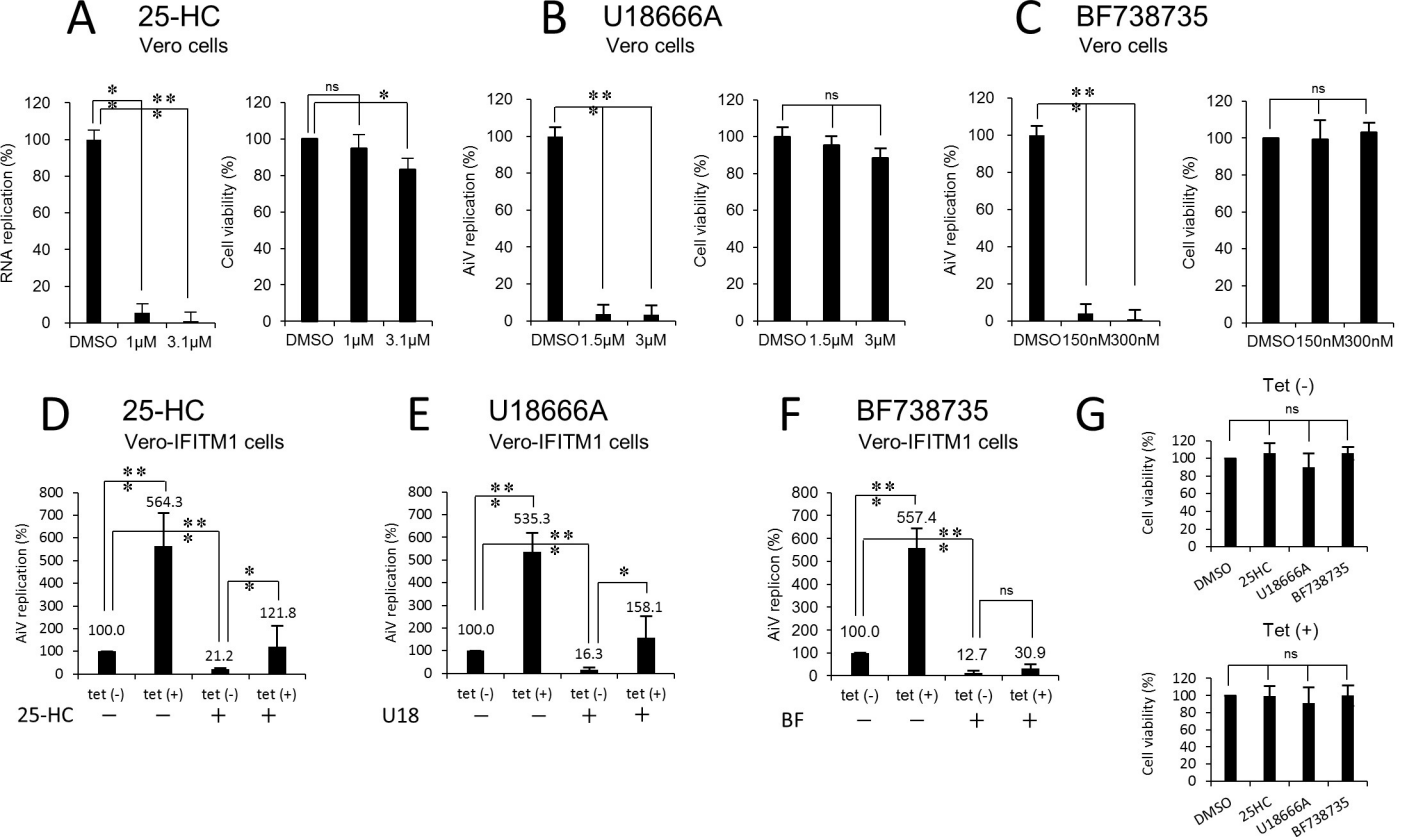
The effect of IFITM1 on cholesterol or PI4P trafficking. (A–F) Vero cells were pretreated with DMSO (as the control) or the indicated concentrations of 25-HC (A), U18666A (B), or BF738735 (C) for 24 h and then transfected with AiV replicon RNA. At 10 h after transfection, the cells were analyzed for luciferase activity. Data were normalized to the DMSO treatment, and cell viability was scored. (D–F) Vero IFITM1 cells were incubated with or without Tet for 48 h and then treated with mock (–) or 1 μM of 25-HC (D), 1.5 μM of U18666A (E), or 150 nM of BF738735 (F). After 24 h, the cells were transfected with AiV replicon RNA, and luciferase activity was determined at 10 h after transfection. (G) Cell viability was measured in parallel. The maximum value obtained for Tet (–) drug-untreated cells was taken as 100%. Data are the mean ± SD of at least three independent experiments. *, *P* < 0.05; **, *P* < 0.001; ***, *P* < 0.0001.

25-HC strongly reduced the replication of the AiV replicon to 6% at 10 h after transfection, even when added to Vero cells at a lower concentration (1 μM; Fig 7A) than that used in a previous report (3.1 μM; [6]). Correspondingly, 25-HC treatment at 1 μM of uninduced Vero-IFITM1 cells reduced replication to 21.2% at 10 h after transfection, without affecting cell viability (Fig 5G), whereas Tet-induced IFITM1 overexpression restored the viral replication to 121.8% of WT level (Fig 7D). A similar experiment using OSW-1, another inhibitor of cholesterol transfer by OSBP, was performed to confirm this finding. As a result, replication, which was reduced to a lesser extent by 43.4% at the optimal concentration of 200 pM, was largely restored to 254% by IFITM1 overexpression (S5A and S5D Fig). Collectively, these data indicate the involvement of IFITM1 in the OSBP-mediated cholesterol transport machinery.

We also focused on PI4P, a lipid produced by PI4KB, which is the driving force of this transport machinery and whose accumulation at the replication sites is essential for AiV replication [4,5]. To investigate whether IFITM1 is concerned with PI4KB-mediated PI4P production, the same analysis was conducted using a PI4KB-specific inhibitor BF738735. The treatment of Vero-IFITM1 cells with only 150 nM BF738735, resulted in an approximately 90% reduction of replication [Fig 7F, Tet (–); BF (+)], whereas no apparent recovery was seen by IFITM1 overexpression [Fig 7F, Tet (+); BF (+)]. This failure to rescue was also observed with another PI4KB inhibitor, GW5074 (S5C and S5F Fig). These data suggest that IFITM1 is unrelated to OSBP/PI4KB-mediated PI4P production at the replication sites.

Given the endosomal localization of IFITM1 [10,11] (Figs 3C, 4E, 6A, 6B, and S1), we additionally confirmed the effect of IFITM1 on endosomal cholesterol transport inhibitor U18666A. As shown in Fig 7B, U18666A impeded replication severely, highlighting that cholesterol efflux from late endosomes is required for viral RNA replication. In U18666A-treated Vero-IFITM1 cells, replication was suppressed significantly by 16.3% [Fig 7E, Tet (–); U18 (+)], but restored by Tet-induced IFITM1 overexpression [Fig 7E, Tet (+); U18 (+)]. A comparable effect was also observed for AY9944, a drug that inhibits endosomal cholesterol metabolism (S5B and S5E Fig). Together with the above results, these data imply that IFITM1 participates in cellular cholesterol transport.

### IFITM1 participates in cholesterol traffic to AiV RNA replication sites

**Fig 8:**
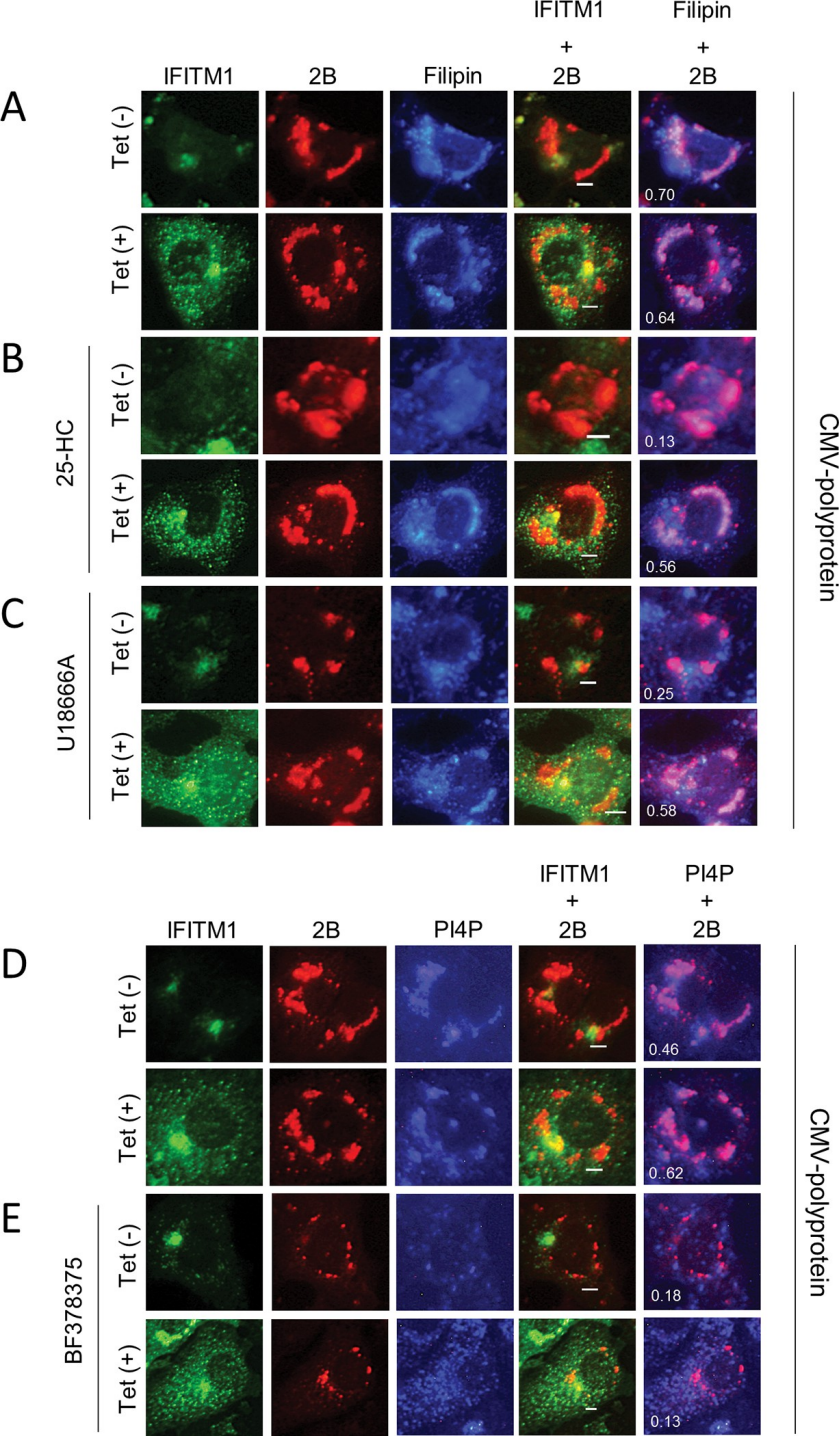
Overexpressed IFITM1 causes cholesterol accumulation at AiV RNA replication sites. (A–E) Vero-IFITM1 cells were incubated with or without Tet for 48 h and then treated with mock (A and D), 1 μM 25-HC (B), 1.5 μM U18666A (C), or 150 nM BF738735 (E), followed by pCMV-polyprotein transfection. At 24 h after transfection, the cells were fixed and stained with filipin III (A–C) or anti-PI4P (D and E), anti-IFITM1 and anti-2B antibodies. Bars, 4 μm. Pearson’s correlation coefficient was measured for individual cells.

To explore the correlation between the rescue of the pharmacologically inhibited AiV RNA replication by IFITM1 and cholesterol distribution at the viral RNA replication sites, we examined filipin distribution at the replication sites by transfecting an AiV polyprotein expression plasmid (pCMV-polyprotein) into Vero-IFITM1 cells in the presence of the cholesterol transport inhibitors. The transient expression of the AiV polyprotein from pCMV-polyprotein in Vero cells has been shown to induce the formation of AiV membranous replication complexes similar to those formed in viral RNA-replicating cells, without the problem of insufficient viral protein production by the inhibitors [5]. In inhibitor-untreated cells (Fig 8A), regardless of the difference in the expression level of IFITM1 depending on the presence or absence of Tet induction, 2B colocalized with filipin, confirming that polyprotein expression in Vero-IFITM1 cells leads to cholesterol accumulation at the replication sites, as reported previously [6]. Overexpressed IFITM1 partially colocalized with 2B [Fig 8A, Tet (+)], as seen in Fig 3D, while it was absent from filipin. This may reflect the results of Fig 6 (at 6 h after replicon RNA transfection), in which IFITM1 is no longer colocalized with filipin as viral protein expression increases.

When IFITM1 expression was not induced in 25-HC- or U18666A-treated cells [Fig 8B and 8C, Tet (–)], 2B-derived fluorescence was dispersed throughout the cytoplasm, and 2B did not colocalize with filipin. However, when IFITM1 was overexpressed, 2B was apparently colocalized with filipin like the control [Fig 8B and 8C, Tet (+)], demonstrating cholesterol accumulation at the replication sites. Similar results were obtained in experiments using OSW-1 or AY9944 (S6B and S6C Fig). Meanwhile, in Fig 8D, the accumulation of PI4P, the PI4KB product, was observed for 2B-containing membrane structures formed in polyprotein-expressing cells, as reported previously [5]. This accumulation was abolished by the PI4KB inhibitor treatment; 2B and filipin were observed as scattered patterns [Fig 8E, Tet (–)], and this inhibitory effect was not restored by IFITM1 overexpression [Fig 8E, Tet (+)]. Together, these results suggest that IFITM1 is involved in cholesterol transport from the ER and late endosomes to the replication sites, resulting in cholesterol accumulation there.

### IFITM1 facilitates the cholesterol transport from the late endosomes to the Golgi

**Fig 9:**
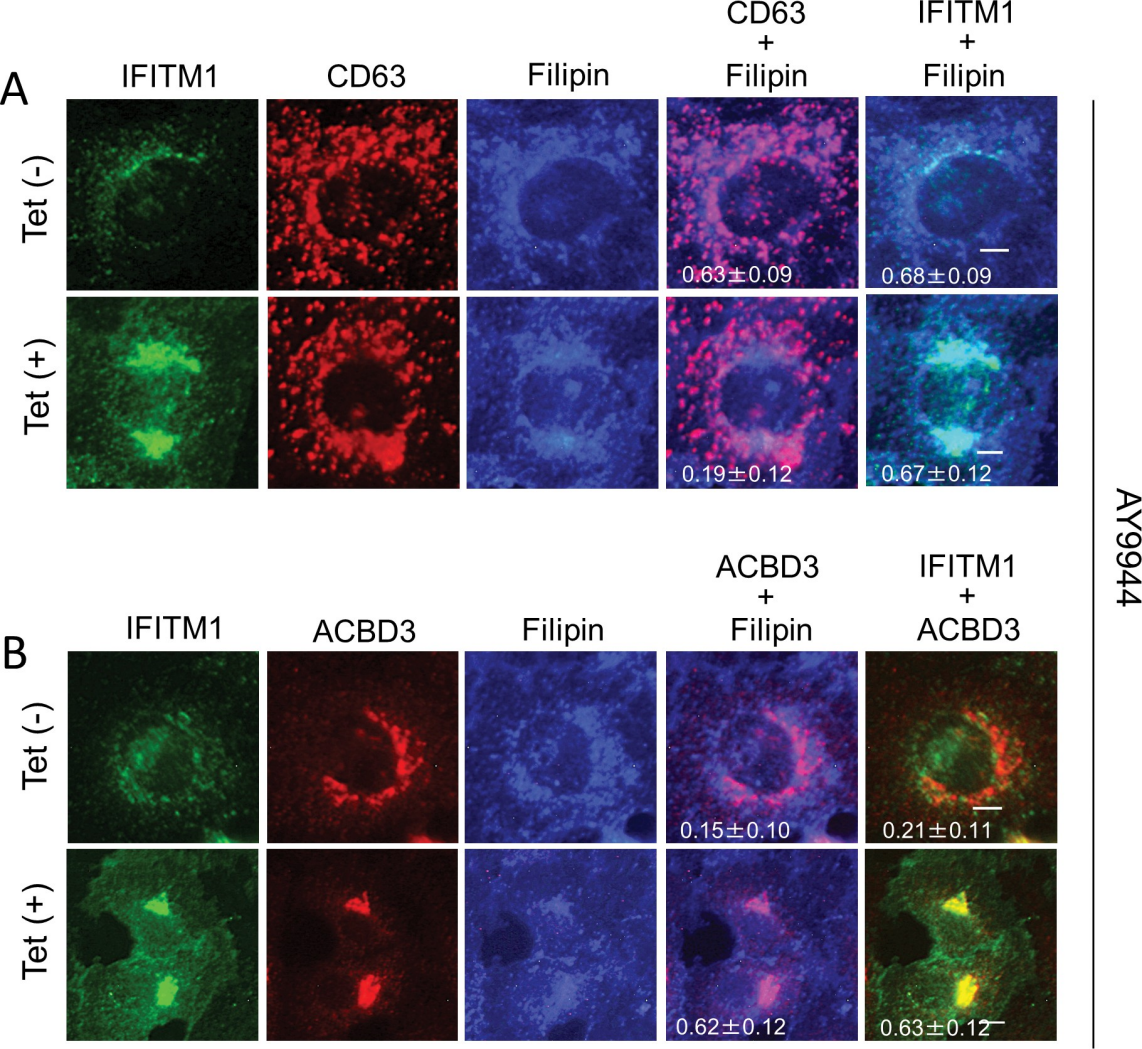
IFITM1 is involved in cholesterol trafficking from late endosomes to the Golgi. (A and B) Vero-IFITM1 cells were incubated with or without Tet for 48 h, and then treated with 3 μM AY9944. After 24 h, the cells were fixed and stained with filipin III and the indicated antibodies. Bars, 4 μm. Pearson correlation coefficient analyses for data were obtained from ≥10 cells. Correlation coefficients are presented as the mean and standard deviation.

Finally, to examine whether viral proteins are required to facilitate late endosomal cholesterol export by the increased expression of IFITM1, Vero-IFITM1 cells treated with AY9944, which is known to induce late endosomal cholesterol accumulation [27,28], were analyzed by immunofluorescence assays. As shown in Fig 9A, when IFITM1 is expressed at the basal level in Tet-uninduced Vero-IFITM1 cells, filipin colocalized with CD63-positive late endosomes, indicating late endosomal cholesterol accumulation [Fig 9A, Tet (–)]. As the expression level of IFITM1 increased by Tet-induction, this degree of colocalization decreased [Fig 9A, Tet (+)], whereas filipin colocalized with ACBD3, suggesting cholesterol transfer to the Golgi [Fig 9B, Tet (+)]. These results suggest that IFITM1 can induce cholesterol transport from late endosomes to the Golgi without any viral proteins.

## Discussion

IFITMs are host restriction factors that inhibit the infection of a number of enveloped RNA viruses, while they are also host factors that promote the infection of some enveloped RNA or DNA viruses [10,18–22,29–33]. Although the role of IFITMs in viral infection has been extensively investigated, its mechanism of action is not yet fully understood. In this study we identified IFITM1 as a factor facilitating genome replication of AiV, a non-enveloped virus, and suggested the involvement of IFITM1 to cholesterol transport to the Golgi or Golgi-derived viral RNA replication sites. Our results provide evidence for a new mechanism of action of IFITM1 on viral replication.

Previous studies have suggested that the viral specificity of IFITM action is associated with the different subcellular localization of IFITM; IFITM1 is located predominantly in the plasma membrane and early endosomes, and IFITM2 and IFITM3 are located in late endosomes and lysosomes [12–14,16,25]. Here we indicated that IFITM1 is partially localized at late endosomes, like IFITM2 and IFITM3, in addition to the plasma membrane and early endosomes. Surprisingly, IFITM1 had the ability to localize to the Golgi when IFITM1 was overexpressed and when viral proteins were underexpressed (Figs 4C–4E, 6C, 9B, and S7B). The late endosomal localization of IFITM1 has also been confirmed in other studies using Vero cells [11,16], A549 cells [10], or HTX cells [26]. On the other hand, an immunogold electron microscopic study showed that IFITM1 is present in the Golgi compartment in IFITM1-HA-expressing A549 cells [14]. Endosomal and Golgi localization of IFITM1 observed in this study may suggest that overexpressed IFITM1, or steady-state level of IFITM1 with the help of viral proteins, has a role in the tethering of both organelles. Consistent with this interpretation, cholesterol accumulated in endosomes was transported to the Golgi in IFITM1-overexpressed cells (Fig 9) and the Golgi-derived replication sites during early stages of viral RNA replication, respectively. Such a transport was not observed at steady-state levels of IFITM1 in uninfected cells (Fig 9). We also observed the localization of IFITM2 and IFITM3 to the Golgi (S8 Fig), but it is not clear whether they have the same function as IFITM1. As for the plasma membrane localization of IFITM1 as previously reported, it was confirmed in overexpressed IFITM1 (Figs 4, S1, S6, and S9), but not in endogenous IFITM1, probably due to low expression levels (Figs 5 and 6).

Our data suggested the role of IFITM1 in OSBP-involved ER-Golgi cholesterol transport. The components of this pathway, OSBP, VAPA, VAPB and SAC1, bound to IFITM1 (Figs 4A, 4B, and S4). In addition, the inhibition of AiV RNA replication by OSBP-mediated cholesterol inhibitors, 25-HC and OSW-1, was recovered by IFITM1 overexpression (Figs 7A, 7D, S5A, and S5D).

Previous study indicated that IFITMs interact with VAPA, and IFITM3–VAPA interaction antagonizes the VAPA–OSBP function, disturbing intracellular cholesterol homeostasis and resulting in the accumulation of endosomal cholesterol [10]. Correspondingly, the increased endosomal cholesterol levels were observed in IFITM1-expressing 549 cells [10]. Our results in this work indicated that IFITM1 expression led to cholesterol accumulation in the Golgi rather than in endosomes (S7 Fig). The reason for this discrepancy is currently unknown but may be due to differences in the cells.

IFITM2 and IFITM3 also bound the proteins involved in OSBP-mediated cholesterol transport (Figs S2A, S2B, and S4) but had little or no effect on enhancing AiV RNA replication (Fig 2A and 2B). Although IFITM1, IFITM2, and IFITM3 share structural domains, the C-terminus of IFITM1 is 13 amino acids longer than that of IFITM2 and IFITM3. This region contains multiple functional motifs whose mutations affect the cellular distribution and antiviral effect, and is involved in its ubiquitination, binding to cellular adaptor protein complex 3 (AP-3), and intracellular trafficking [19,25,26,34]. Thus, these motifs may play a role in AiV replication. Another possibility is that IFITM1, IFITM2, and IFITM3 may exert different effects on AiV replication, as reported for the antiviral roles of IFITM2 and IFITM3 in African swine fever virus infection [11].

In summary, this study described the role of IFITM in AiV RNA replication. Early in infection, steady-state level of IFITM1, together with viral proteins, would enable efficient RNA replication by transporting cholesterol to the AiV RNA replication sites. Also, overexpressed IFITM1 would be able to transport cholesterol to the Golgi. Overexpression of IFITM1 will be induced by IFN produced upon AiV infection. However, the RNA replication was inhibited by IFN treatment (Fig 1), suggesting that the the ability of IFITM1 to upregulate AiV replication is not sufficient to overcome the antiviral response provided by the various ISGs. IFITM1 may function immediately after infection, before an IFN response is induced, thereby upregulating viral RNA replication. In fact, IFITM1 was localized at the AiV RNA replication sites early in infection, suggesting its involvement in cholesterol accumulation there. (Figs 5A, 5B, 6C, 6D, 8A–8C, and S6A–S6C). Alternatively, AiV may suppress the effects of IFN produced in response to viral infection, such as EV-71 and RV-14 [35,36], thus allowing steady-state levels of IFITM1 to function. Our findings are considered to be an important step in clarifying the unexplained function of IFITM1.

## Materials and methods

### Cells and virus

293T, HeLa, or Vero cells were grown in Eagle’s minimum essential medium (EMEM) containing 5% fetal bovine serum (FBS), appropriate antibiotics (10 μg/ml fungizone and 200 μg/ml kanamycin), and 2 mM L-glutamine at 37°C. Vero, HeLa, and 293T cells expressing each IFITM by Tet induction were generated using pcDNA4/TO containing each IFITM and pcDNA6/TR (T-REx system; Invitrogen) according to the manufacturer’s protocol. The stable cell lines were grown in 5% FBS-containing EMEM supplemented with 15 μg/ml blasticidin and 300 μg/ml Zeocin, and the expression of each IFITM was induced by adding 1 μg/ml Tet into the culture medium. AiV (strain A846/88) was kindly provided by Dr. Teruo Yamashita at the Aichi Prefectural Institute of Public Health (Aichi, Japan) and grown in Vero cells [2].

### Plasmid constructs

The construction of pBIND-2B, pBIND-2BC, pBIND-2C, pBIND-3A, pBIND-3AB, pACT-2B, pACT-2BC, pACT-2C, pACT-3A, pACT-3AB, pCI-HA-2B, pCI-HA-2BC, pCI-HA-2C, pCI-HA-3A, pCI-HA-3AB, pCI-HA-ACBD3, pCI-HA-PI4KB, pCI-HA-OSBP, pCI-HA-VAPA, pCI-HA-VAPB, pCI-HA-SAC1, and pCMV-polyprotein was described previously [6,37]. The coding regions of IFITM1, IFITM2, and IFITM3 were amplified by PCR using primer pairs containing *MluI*–*EcoRV* sites from cDNA clones (OriGene; IFITM1: RC201617, IFITM2: RC202067, IFITM3: RC201635), respectively, and then inserted into the same sites of pACT and pBIND, resulting in pACT-IFITM1, pACT-IFITM2, pACT-IFITM3, pBIND-IFITM1, pBIND-IFITM2, and pBIND-IFITM3. For expressing FLAG-fused IFITM1, IFITM2, or IFITM3, each MluI-NotI fragment derived from the pBIND constructs was cloned into the same sites of pCI (Promega) containing a FLAG tag sequence between the *NheI*–*MluI* sites, generating pCI-FLAG-IFITM1, pCI-FLAG-IFITM2, and pCI-FLAG-IFITM3. To create stable cell lines, pcDNA-IFITM1, -IFITM2, and -IFITM3 were generated by ligating the *Not*I–*Bam*HI fragments of pBIND-IFITM1, -IFITM2, and -IFITM3, respectively, to pcDNA4/TO (Invitrogen).

### Antibodies and reagents

Rabbit polyclonal anti-IFITM1 antibody was purchased from Atlas antibodies. Rabbit polyclonal anti-IFITM2 antibody was obtained from Proteintech. This IFITM2 antibody has been reported to cross-react with IFITM3 [38]. Therefore, we denote the IFITM protein recognized by anti-IFITM2 as IFITM2/3. Rabbit polyclonal anti-IFITM3 antibody was from ABGENT. Guinea pig polyclonal anti-2A antibody was raised against His-tagged 2A expressed in *Escherichia coli* (Medical & Biological Laboratories Co., Ltd. [MBL], Nagoya, Japan). Mouse monoclonal anti-EEA and anti-PI4KB antibodies were from BD Biosciences. Mouse monoclonal anti-CD63 antibody was from Novus Biologicals. Mouse monoclonal anti-ACBD3 was from Sigma. Mouse monoclonal anti-OSBP was from Invitrogen. Guinea pig polyclonal anti-2B, anti-2C, anti-3A, anti-VAPA and anti-VAPB antibodies, rabbit polyclonal anti-FLAG antibody, sheep polyclonal anti-TGN46, mouse monoclonal IgM anti-PI4P antibody, and mouse monoclonal anti-HA were described previously [4,6]. Alexa Fluor 488-, 594- or 350-conjugated anti-mouse IgG anti-mouse IgM, anti-rabbit IgG or anti-guinea pig IgG were purchased from Molecular Probes. AMCA-conjugated anti-guinea pig IgG was from Millipore. Horseradish peroxidase-conjugated antibody was from Invitrogen. Filipin III and 25-HC were described previously [6]. U18666A and AY9944 were obtained from Cayman Chemical. GW5074 was from Sigma. BF738735 was from Med Chem Express. OSW-1 was from Toronto Research Chemicals.

### si RNA transfection

HeLa cells were transfected with 60 nM siRNA using Lipofectamine 2000 reagent (Invitrogen) according to the manufacturer’s protocol. Control siRNA (siGENOME Non-Targeting Control siRNA #3), and siRNA against IFITM1 (siGENOME Human IFITM1 siRNA SMARTpool), IFITM2 (siGENOME Human IFITM2 siRNA SMARTpool), and IFITM3 (siGENOME Human IFITM3 siRNA SMARTpool) were purchased from Dharmacon.

### Immunofluorescent staining

Cells were cultured on 8-well glass slide. Transfection of HA- and FLAG-tagged plasmids and pCMV-polyprotein were performed using TransIT-2020 (Mirus Bio). AiV infection was performed for 1 h at 37°C. Cells were fixed with 4% paraformaldehyde in PBS for 45min and Vero cells were then permeabilized with 0.5% triton X-100 in phosphate-buffered saline (PBS) for 5min, Vero-IFITM1 cells and Hela-IFITM1 cells with 0.2% saponin in PBS containing 3% bovine serum albumin (BSA) for 30 min or 20 μM digitonin in PBS for 5 min (for PI4P staining). After fixation and permeabilization, immunostaining of cells was performed as described previously [4,6]. Images were obtained using a fluorescence microscope, BZ-8000, BZ-9000, BZ-X800 (Keyence). Pearson’s correlation coefficient was measured for individual cells using Coloc 2 Fiji’s plugin.

### Replicon assay

Replicon RNA, an in vitro transcript from full-length AiV cDNA in which the capsid-coding region is replaced by a firefly luciferase gene, was synthesized using T7 RiboMAX express large scale RNA production system (Promega) as described previously [39,40]. Cells were all prepared in 48 well-plates and 125 ng/well of replicon RNA was transfected using Lipofectin (Invitrogen) according to the manufacturer’s protocol. At the indicated time points after transfection, cell lysates in 1× Passive lysis buffer (Promega) were harvested, and luciferase activity was measured using a Luciferase assay system (Promega) with Lumat luminometer (Berthold).

### Immunoprecipitation and immunoblotting

Immunoprecipitation assays and immunoblotting were performed as described earlier [4]. Densitometric analyses were performed using the software ImageJ.

### Mammalian two-hybrid assays (M2H)

The M2H was performed as described previously using a Checkmate M2H system (Promega) [37]. For example, to study the interaction between proteins X and Y, Luc activity obtained from cotransfection with the pACT-X:pBIND-Y pair was compared to the higher values from cotransfection with the pACT:pBIND-Y pair and cotransfection with the pACT-X:pBIND pair, and Luc activity from cotransfection with pACT-Y:pBIND-X pair was compared to the higher values from cotransfection with the pACT:pBIND-X pair and cotransfection with the pACT-Y:pBIND pair.

## Supporting information

S1 Fig Colocalization of IFITM1 with the endosomal markers EEA1, CD63, and LBPA. Tet-inducible Vero-IFITM1 (left) or HeLa-IFITM1 (right) cells were cultured with or without Tet. After 72 h, the cells were fixed and stained with anti-IFITM and anti-EEA1 (top), anti-CD63 (middle), or anti-LBPA (bottom) antibodies, as indicated. Bars, 4 μm.
(TIF)

S2 Fig Coimmunoprecipitation analysis of the interactions of IFITM1 with viral proteins and Golgi proteins. (A) The same experiment as in Fig 3B was perfomed. (B) The experiment was performed by replacing the tags used in Fig 4B.
(TIF)

S3 Fig OSBP colocalizes with the endosomal markers EEA1 and CD63 in cells with low 3A expression. Vero cells were electroporated with replicon RNA. After 4h, the cells were fixed and labeled with the indicated antibodies. Bars, 4 μm.
(TIF)

S4 Fig Interaction of IFITMs with the ER proteins, VAPA, VAPB, and SAC1. (A) M2H analyses were performed to determine the interactions between IFITM1, IFITM2, or IFITM3 and VAPA (left), VAPB (middle), or SAC1 (right), and the results are shown as described in Fig 3A. Data are the mean ± SD of at least three independent experiments. (B) 293T cells transfected with the indicated combination of FLAG-tagged or HA-tagged constructs were subjected to immunoprecipitation, followed by immunoblotting with the indicated antibodies.
(TIF)

S5 Fig Restoration of AiV RNA replication by overexpressed IFITM1 in cells treated with different cholesterol trafficking inhibitors. (A) Vero cells were pretreated with DMSO (control) or the indicated concentrations of OSW-1 (A), AY9944 (B), or GW5074 (C) for 24h, followed by transfection with AiV replicon RNA. At 10 h after transfection, luciferase activity and cell viability were measured. Data were normalized against the DMSO control. (D–G) Vero-IFITM1 cells were incubated with Tet (–) or Tet (+) for 48 h, and then treated with mock (–) or 200 pM OSW-1 (D), 3 μM AY9944 (E), or 16 μM GW5074 (F) for 24 h, followed by transfection with AiV replicon RNA. At 10 h after transfection, the luciferase activity and cell viability (G) were measured. The maximum value obtained for Tet (–) drug-untreated cells was taken as 100%. Data are the mean ± SD of at least three independent experiments. *, *P* < 0.05; **, *P* < 0.001; ***, *P* < 0.0001.
(TIF)

S6 Fig Verification of cholesterol accumulation at AiV RNA replication sites by overexpressed IFITM1 in cells treated with different cholesterol trafficking inhibitors. (A–E) Vero-IFITM1 cells were cultured with or without Tet for 48 h, and then mock treated (A and D) or treated with 200 pM OSW-1 (B), 3 μM AY9944 (C), or 16 μM GW5074 (E), followed by pCMV-polyprotein transfection. After 24 h, the cells were immunolabeled with filipin III (A–C) or anti-PI4P (D and E), anti-IFITM1 and anti-2B antibodies. Bars, 4 μm.
(TIF)

S7 Fig Overexpressed IFITM1 alters the localization of filipin III from late endosomes to the Golgi. (A and B) Vero-IFITM1 cells were incubated with or without Tet. After 72h, the cells were fixed and then stained with filipin III and the indicated antibodies. Bars, 4 μm.
(TIF)

S8 Fig IFITM2 and IFITM3 bind to the Golgi proteins ACBD3, PI4KB, and OSBP. (A) M2H analyses were performed to determine the interactions between IFITM2/IFITM3 and ACBD3 (left), PI4KB (middle), or OSBP (right), and the results are shown as described in Fig 3A. Data are the mean ± SD of at least three independent experiments. (B) 293T cells transfected with the indicated combination of FLAG-tagged or HA-tagged constructs were subjected to immunoprecipitation, followed by immunoblotting with the indicated antibodies. (C) Vero cells expressing FLAG-IFITM2, or FLAG-IFITM3 were immunostained with anti-FLAG and anti-ACBD3 (left), anti-PI4KB (middle), or anti-OSBP (right) antibodies. (D) HeLa-IFITM2 (left) or HeLa-IFITM3 (right) cells were incubated with or without Tet. After 72 h, the cells were fixed and then stained with anti-TGN46 and anti-IFITM2 (left), or anti-IFITM3 (right) antibodies. Bars, 4 μm.
(TIF)

S9 Fig Subcellular distribution of IFITM1 according to expression level. Vero-IFITM1 cells were incubated with Tet for 72 h, and the cells were then fixed and permeated with 0.2% saponin. After permeabilization, the cells were immunolabeled with anti-IFITM1 antibody. Bars, 4 μm.
(TIF)

S1 Dataset Data for Fig 1A–1C. (XLSX)

S2 Dataset Data for Fig 2A–2D, and 2F. (XLSX)

S3 Dataset Data for Figs 3A and 4A. (XLSX)

S4 Dataset Data for Fig 7A–7G. (XLSX)

S5 Dataset Data for Figs 3C, 3D, 4C–4E, 5A, 5B, 6A–6D, 9A, and 9B. (XLSX)
