# Supplementary figures and images for: IFITM1 enhances nonenveloped viral RNA replication by facilitating cholesterol transport to the Golgi
Source: PLoS Pathog. 2023 May 30;19(5):e1011383. doi: 10.1371/journal.ppat.1011383 (PMC10256215; doi:10.1371/journal.ppat.1011383)

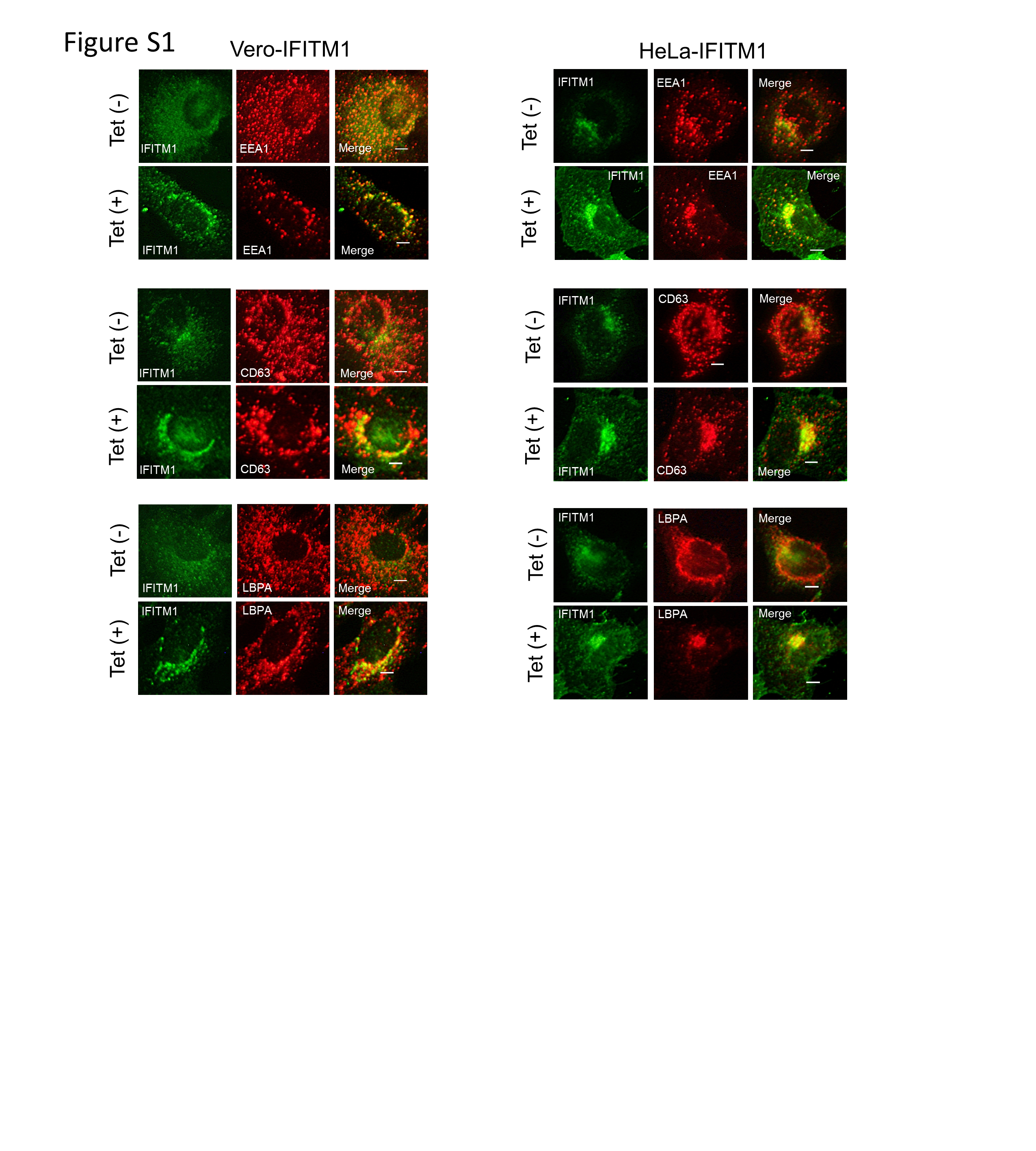

Supplement: S1 Fig — Tet-inducible Vero-IFITM1 (left) or HeLa-IFITM1 (right) cells were cultured with or without Tet. After 72 h, the cells were fixed and stained with anti-IFITM and anti-EEA1 (top), anti-CD63 (middle), or anti-LBPA (bottom) antibodies, as indicated. Bars, 4 μm. (TIF) [file ppat.1011383.s001.tif]

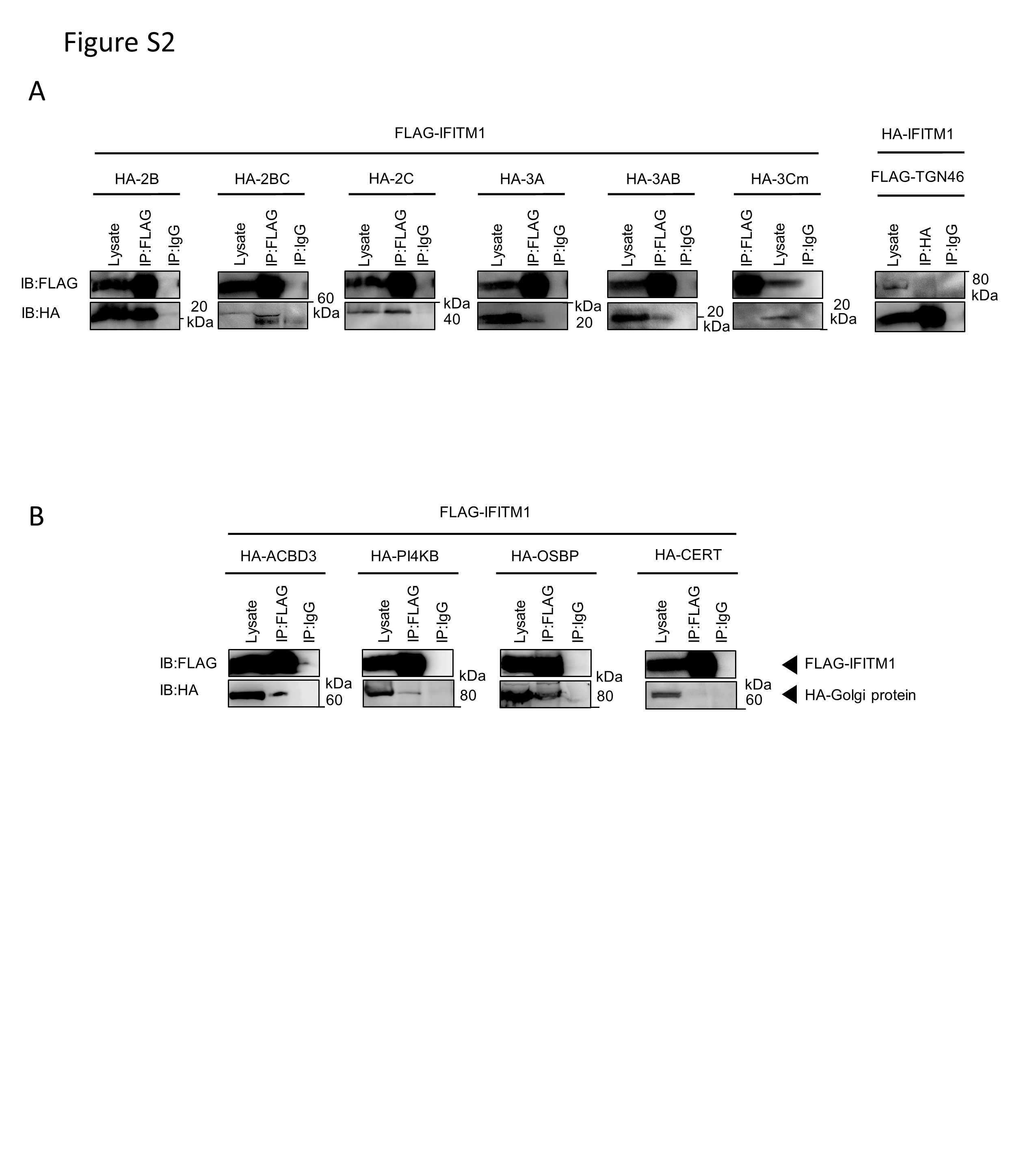

Supplement: S2 Fig — (A) The same experiment as in Fig 3B was perfomed. (B) The experiment was performed by replacing the tags used in Fig 4B. (TIF) [file ppat.1011383.s002.tif]

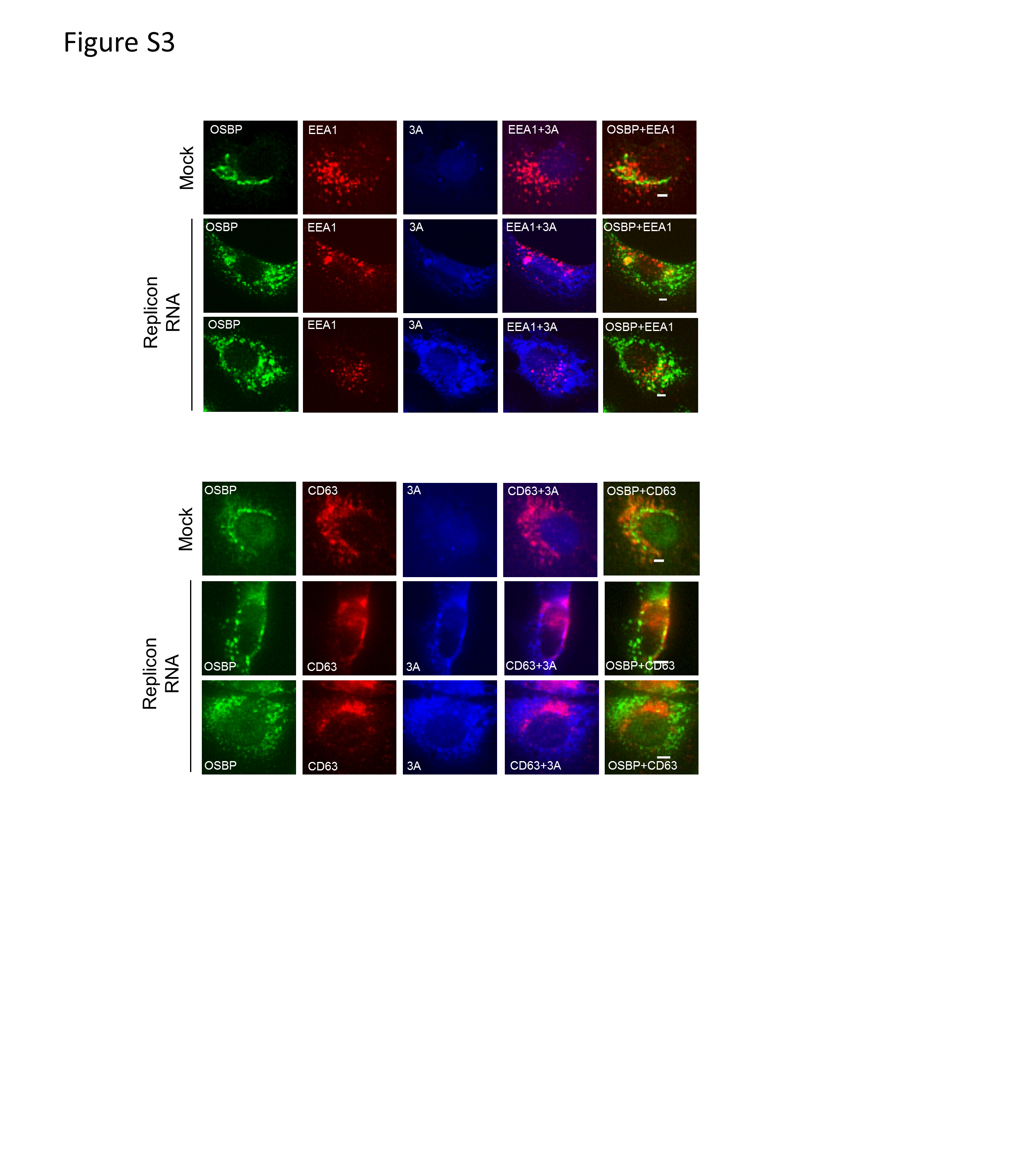

Supplement: S3 Fig — Vero cells were electroporated with replicon RNA. After 4h, the cells were fixed and labeled with the indicated antibodies. Bars, 4 μm. (TIF) [file ppat.1011383.s003.tif]

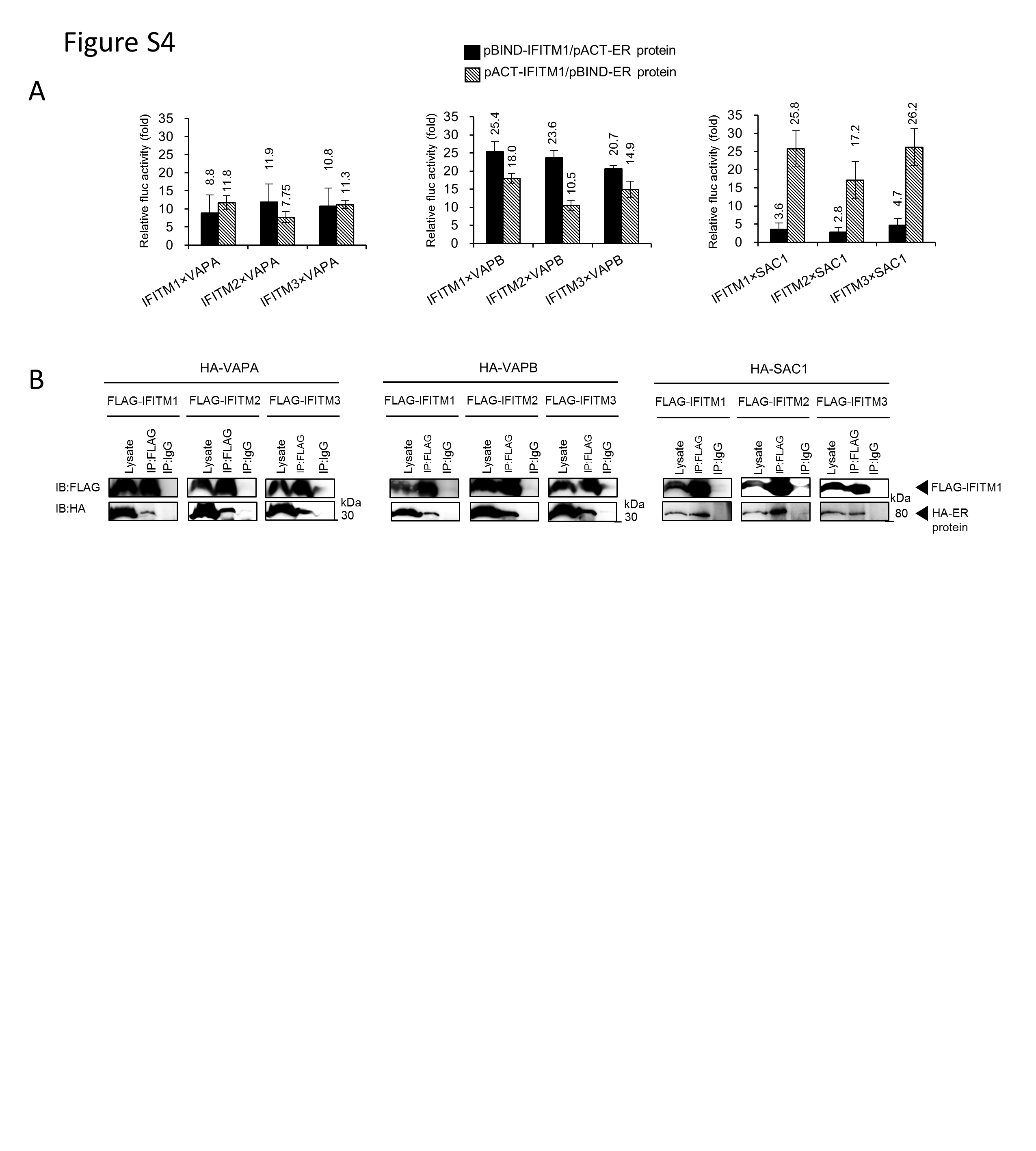

Supplement: S4 Fig — (A) M2H analyses were performed to determine the interactions between IFITM1, IFITM2, or IFITM3 and VAPA (left), VAPB (middle), or SAC1 (right), and the results are shown as described in Fig 3A. Data are the mean ± SD of at least three independent experiments. (B) 293T cells transfected with the indicated combination of FLAG-tagged or HA-tagged constructs were subjected to immunoprecipitation, followed by immunoblotting with the indicated antibodies. (TIF) [file ppat.1011383.s004.tif]

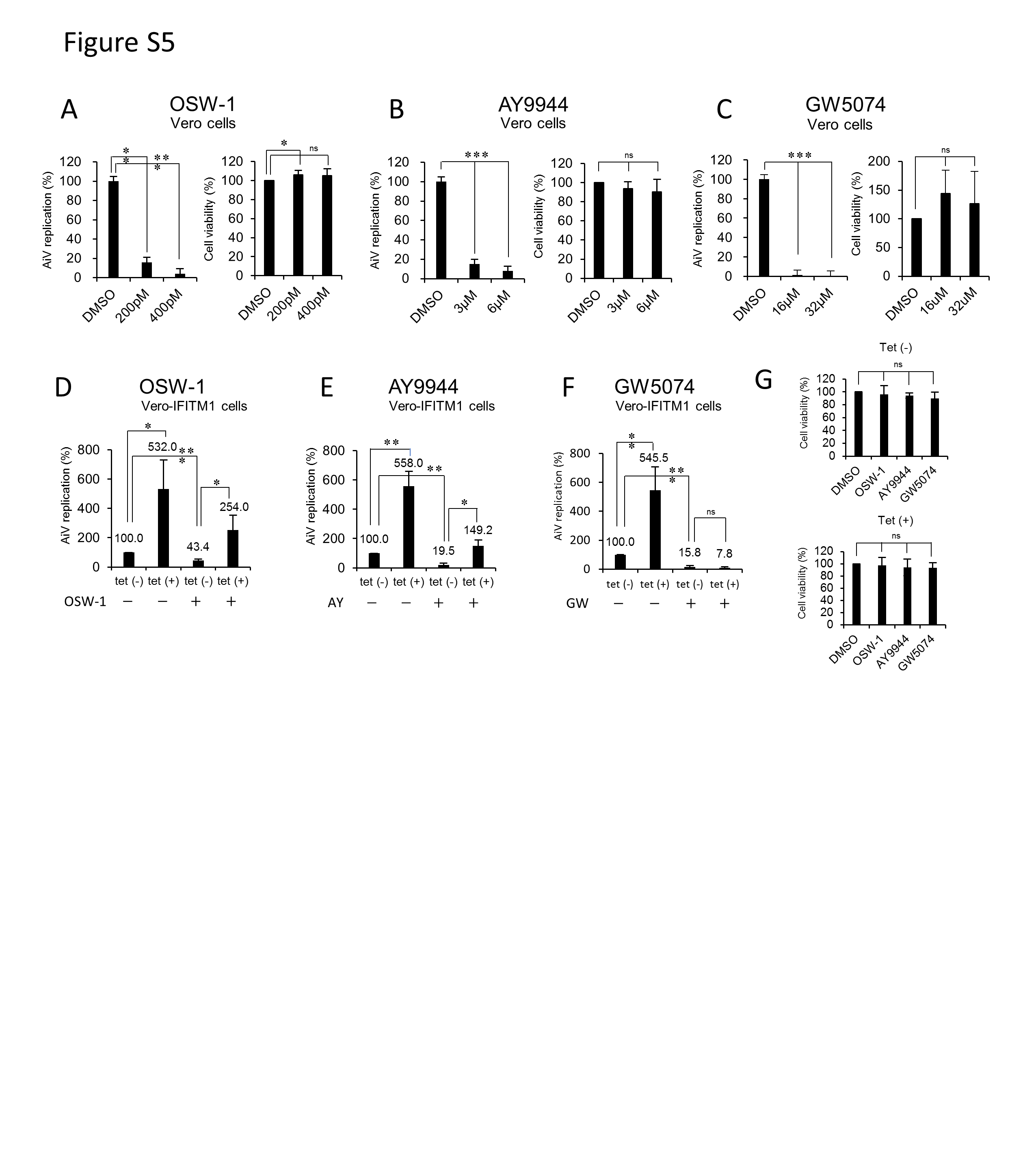

Supplement: S5 Fig — (A) Vero cells were pretreated with DMSO (control) or the indicated concentrations of OSW-1 (A), AY9944 (B), or GW5074 (C) for 24h, followed by transfection with AiV replicon RNA. At 10 h after transfection, luciferase activity and cell viability were measured. Data were normalized against the DMSO control. (D–G) Vero-IFITM1 cells were incubated with Tet (–) or Tet (+) for 48 h, and then treated with mock (–) or 200 pM OSW-1 (D), 3 μM AY9944 (E), or 16 μM GW5074 (F) for 24 h, followed by transfection with AiV replicon RNA. At 10 h after transfection, the luciferase activity and cell viability (G) were measured. The maximum value obtained for Tet (–) drug-untreated cells was taken as 100%. Data are the mean ± SD of at least three independent experiments. *, P < 0.05; **, P < 0.001; ***, P < 0.0001. (TIF) [file ppat.1011383.s005.tif]

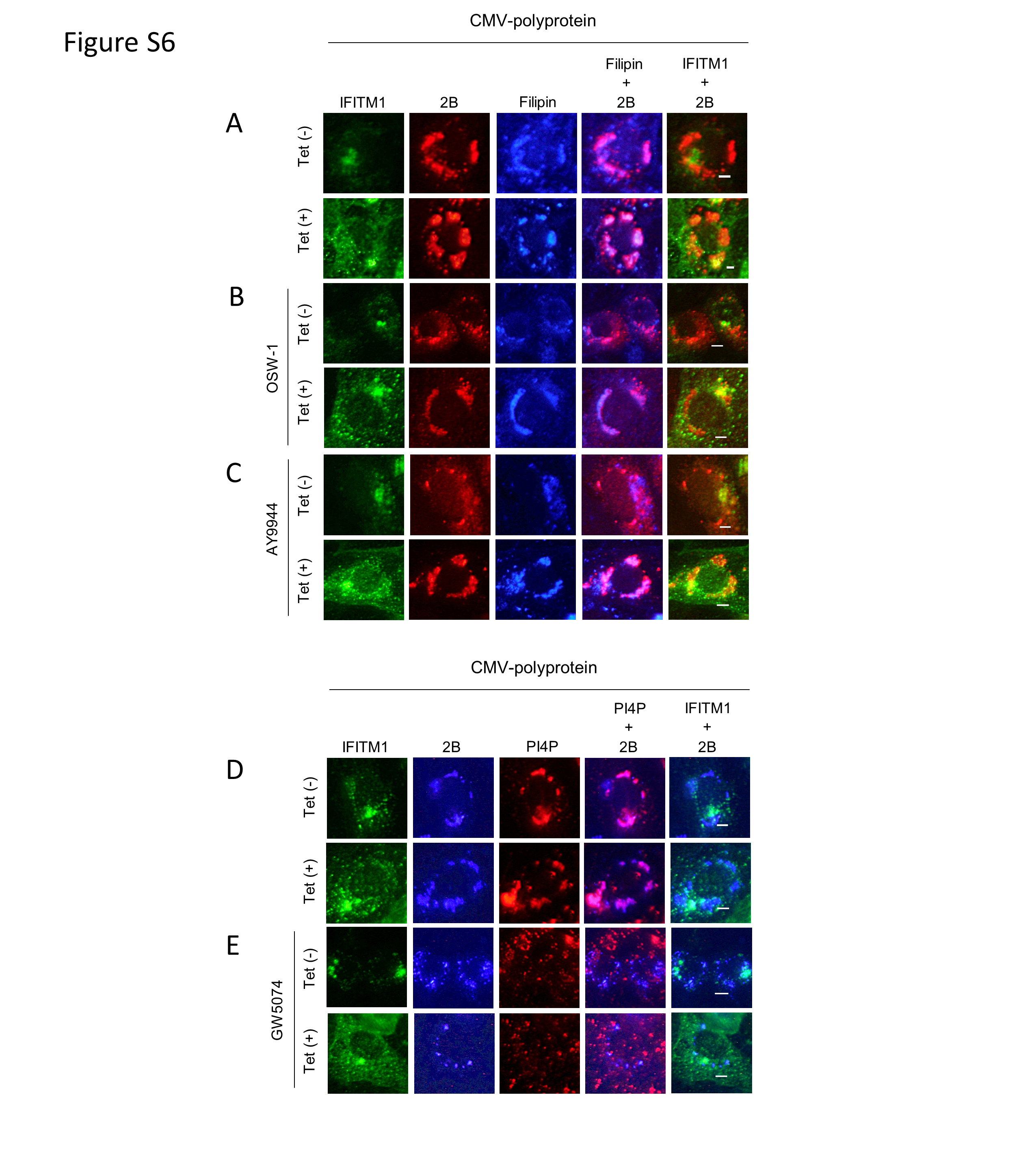

Supplement: S6 Fig — (A–E) Vero-IFITM1 cells were cultured with or without Tet for 48 h, and then mock treated (A and D) or treated with 200 pM OSW-1 (B), 3 μM AY9944 (C), or 16 μM GW5074 (E), followed by pCMV-polyprotein transfection. After 24 h, the cells were immunolabeled with filipin III (A–C) or anti-PI4P (D and E), anti-IFITM1 and anti-2B antibodies. Bars, 4 μm. (TIF) [file ppat.1011383.s006.tif]

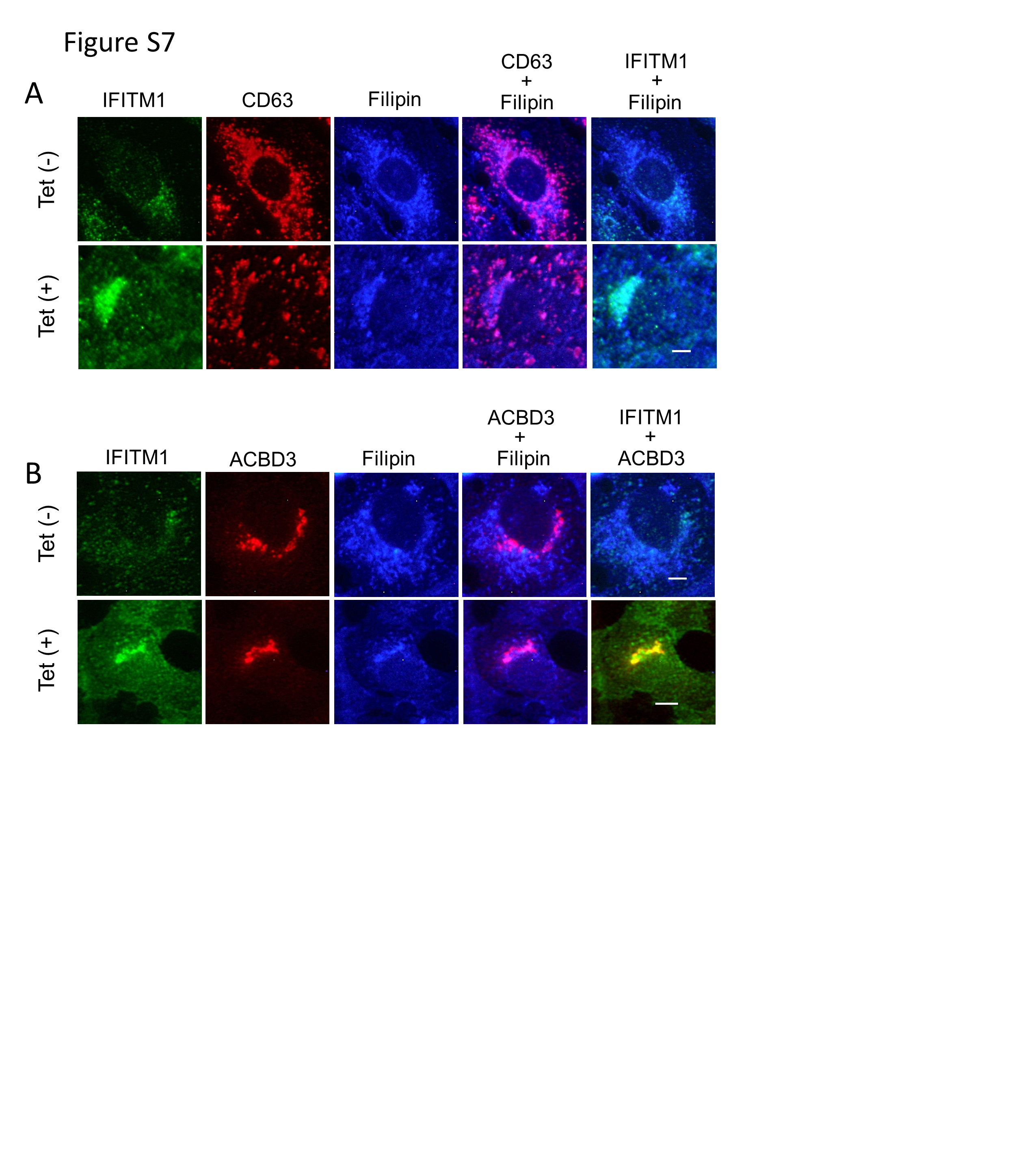

Supplement: S7 Fig — (A and B) Vero-IFITM1 cells were incubated with or without Tet. After 72h, the cells were fixed and then stained with filipin III and the indicated antibodies. Bars, 4 μm. (TIF) [file ppat.1011383.s007.tif]

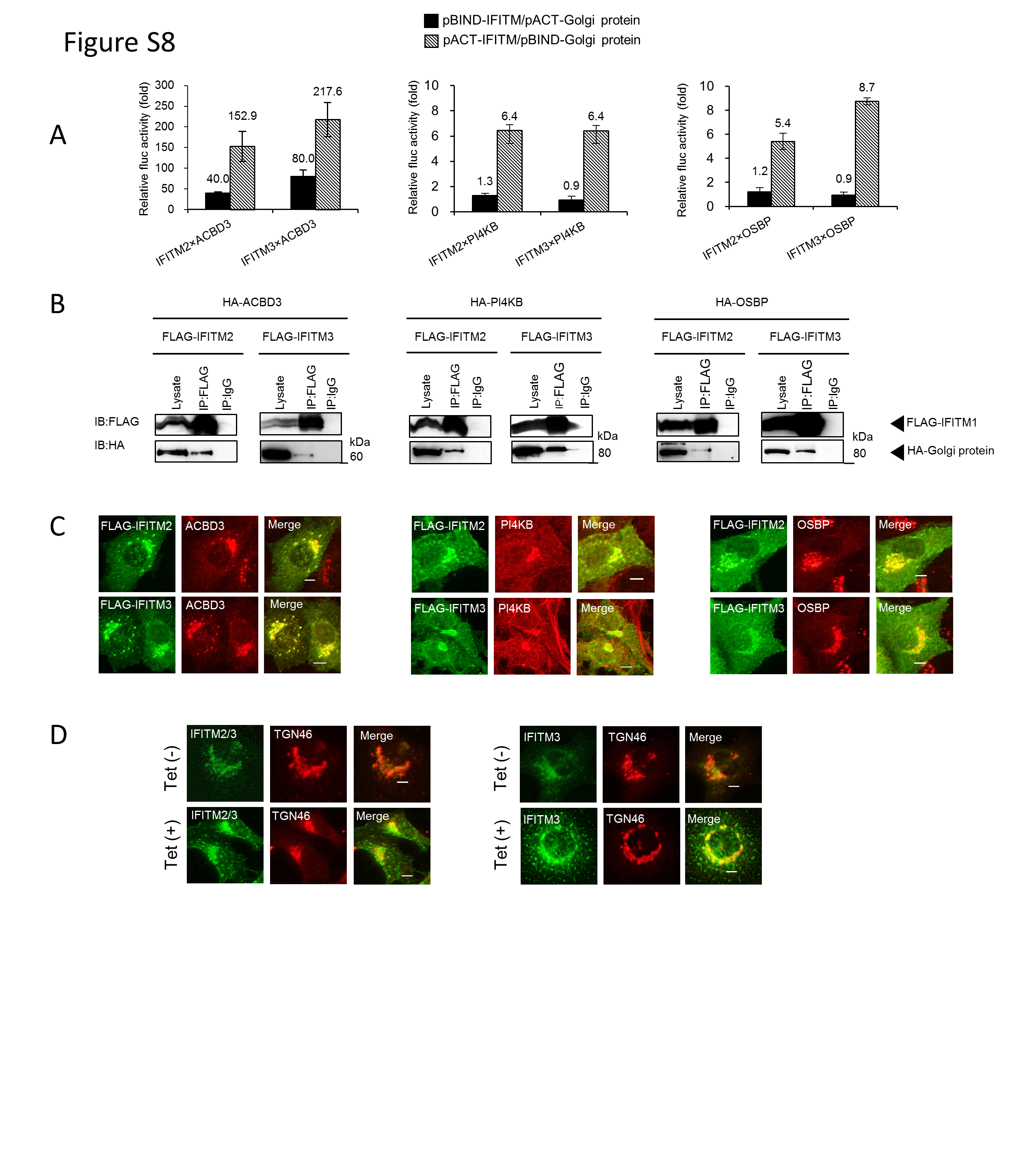

Supplement: S8 Fig — (A) M2H analyses were performed to determine the interactions between IFITM2/IFITM3 and ACBD3 (left), PI4KB (middle), or OSBP (right), and the results are shown as described in Fig 3A. Data are the mean ± SD of at least three independent experiments. (B) 293T cells transfected with the indicated combination of FLAG-tagged or HA-tagged constructs were subjected to immunoprecipitation, followed by immunoblotting with the indicated antibodies. (C) Vero cells expressing FLAG-IFITM2, or FLAG-IFITM3 were immunostained with anti-FLAG and anti-ACBD3 (left), anti-PI4KB (middle), or anti-OSBP (right) antibodies. (D) HeLa-IFITM2 (left) or HeLa-IFITM3 (right) cells were incubated with or without Tet. After 72 h, the cells were fixed and then stained with anti-TGN46 and anti-IFITM2 (left), or anti-IFITM3 (right) antibodies. Bars, 4 μm. (TIF) [file ppat.1011383.s008.tif]

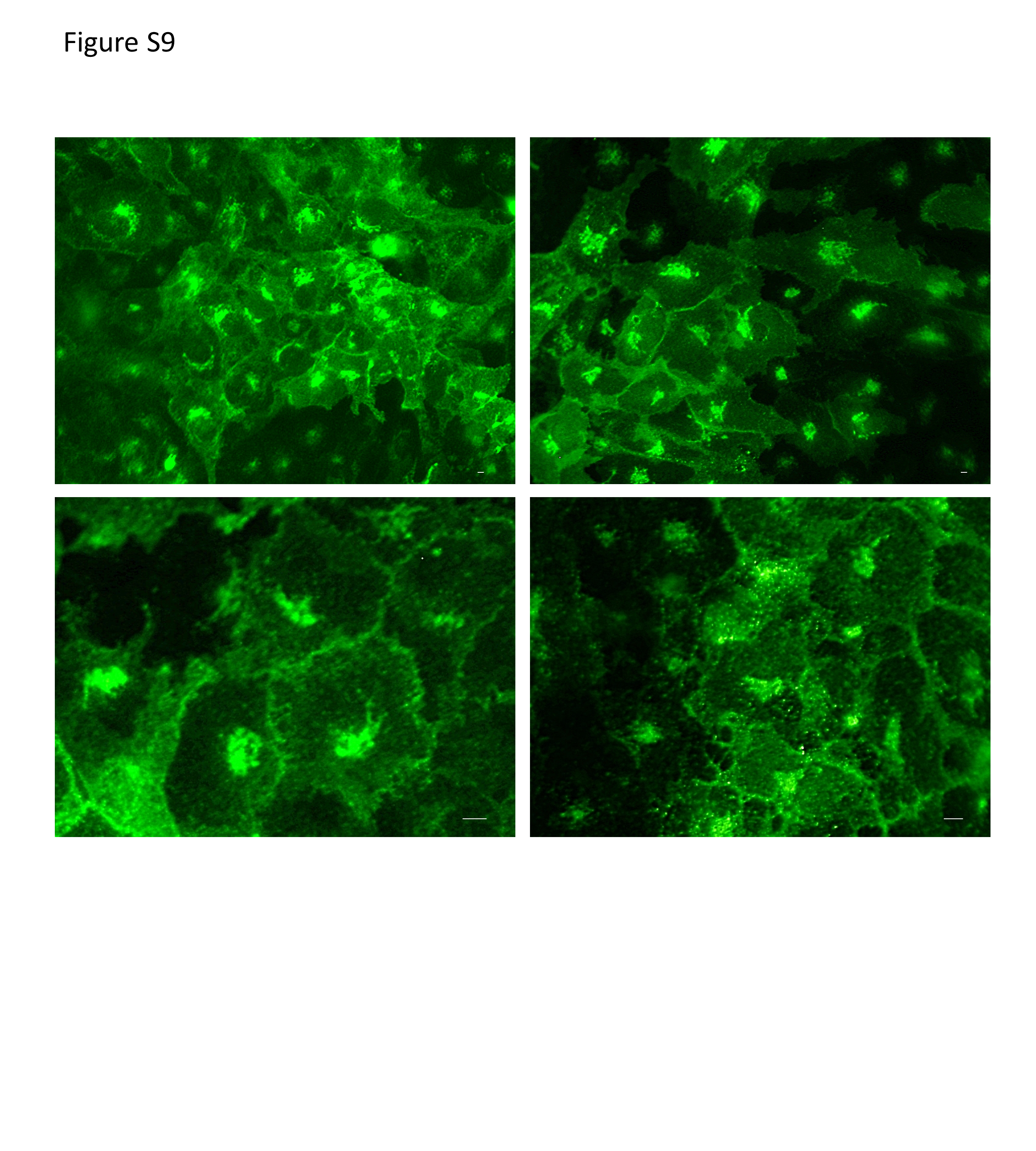

Supplement: S9 Fig — Vero-IFITM1 cells were incubated with Tet for 72 h, and the cells were then fixed and permeated with 0.2% saponin. After permeabilization, the cells were immunolabeled with anti-IFITM1 antibody. Bars, 4 μm. (TIF) [file ppat.1011383.s009.tif]
